# Multifunctional 3D-Printed Alginate Emulgel Patches Incorporating Plant Extracts for Potential Burn Wound Applications

**DOI:** 10.3390/gels12060541

**Published:** 2026-06-17

**Authors:** Roxana Colette Sandulovici, Ion Mircioiu, Mariana Panțuroiu, Corneliu Dan Blendea, Mirela Claudia Rîmbu, Daniel Cord, Carmen Elisabeta Manea, Carmen Marinela Mihăilescu, Mirela Antonela Mihăilă, Iulian Sârbu, Horia Sebastian Iliescu, Manuel Ovidiu Amzoiu, Adina Boldeiu, Vasilica Țucureanu, Oana Brîncoveanu, Luiza Mădălina Cima, Mona Luciana Gălățanu

**Affiliations:** 1 Faculty of Pharmacy, Titu Maiorescu University, 040314 Bucharest, Romania; roxana.sandulovici@prof.utm.ro (R.C.S.); ion.mircioiu@prof.utm.ro (I.M.); mirela.rimbu@prof.utm.ro (M.C.R.); daniel.cord@prof.utm.ro (D.C.); carmen.manea@prof.utm.ro (C.E.M.); carmen.mihailescu@prof.utm.ro (C.M.M.); mirela.mihaila@prof.utm.ro (M.A.M.); iulian.sarbu@prof.utm.ro (I.S.); horia.iliescu@s.utm.ro (H.S.I.); luiza.cima@prof.utm.ro (L.M.C.); luciana.galatanu@prof.utm.ro (M.L.G.); 2 Faculty of Medicine, Titu Maiorescu University, 040314 Bucharest, Romania; 3 Horia Hulubei National Institute for R&D in Physics and Nuclear Engineering (IFIN-HH), Reactorului 30 St., 077125 Magurele, Romania; 4 National Institute for Research and Development in Microtehnologies (IMT), 077190 Bucharest, Romania; adina.boldeiu@imt.ro (A.B.); vasilica.tucureanu@imt.ro (V.Ț.); oana.brincoveanu@imt.ro (O.B.); 5 Doctoral School, University of Medicine and Pharmacy of Craiova, 200349 Craiova, Romania; 6 Stefan S. Nicolau Institute of Virology, Mihai Bravu St, 030304 Bucharest, Romania; 7 Faculty of Pharmacy, University of Medicine and Pharmacy of Craiova, 200349 Craiova, Romania; manuel.amzoiu@umfcv.ro

**Keywords:** alginate, emulgel, 3D printing, burn wound dressing, plant extracts, polyphenols, antioxidant activity, HaCaT cells, burn wound applications

## Abstract

Multifunctional dressings capable of maintaining a moist environment, supporting tissue regeneration, and delivering bioactive compounds are increasingly being explored as promising strategies for burn wound management. In this study, alginate-based emulgel patches incorporating hydrophilic and lipophilic plant extracts were developed by extrusion-based 3D printing as potential topical systems for burn wound applications. The formulation included sodium alginate, hyaluronic acid, and hydroglyceric extracts of *Calendula officinalis*, *Matricaria chamomilla*, and *Plantago major*, as well as oily extracts of *Hippophae rhamnoides* and *Hypericum perforatum*. The emulgel was evaluated for pH, rheological behaviour, spreadability, physical stability, apparent hydrodynamic size distribution, zeta potential, total polyphenol content, and antioxidant activity. Following Ca^2+^-induced crosslinking, uniform and flexible 3D-printed patches were obtained and further characterised for pharmacotechnical, physicochemical, structural, functional, and biological properties. The emulgel exhibited suitable characteristics for extrusion-based printing, while the resulting patches showed good dimensional uniformity, flexibility, swelling capacity, water vapour transmission, and surface pH compatible with topical application. FTIR, DLS, SEM, and SEM–EDX analyses supported the formation of a Ca^2+^-crosslinked alginate network and confirmed the presence of structurally heterogeneous domains with homogeneous calcium distribution. The patches retained plant-derived bioactive compounds, with a total polyphenol content of 0.2878 ± 0.016 mg GAE/g hydrated patch, and showed improved antioxidant activity compared with the corresponding emulgel. In vitro release studies indicated the time-dependent diffusion of polyphenols over 24 h, with cumulative release reaching 64.42%. The patches also exhibited a water vapour transmission rate of 1270 ± 93 g/m^2^/24 h, indicating adequate moisture regulation. HaCaT cell viability remained above 90% at lower tested concentrations, demonstrating a favourable biocompatibility profile. Overall, the developed 3D-printed alginate emulgel patches represent promising multifunctional systems for potential burn wound management and warrant further preclinical investigation.

## 1. Introduction

Burns and chronic wounds represent a major healthcare challenge. Their complex healing processes, high susceptibility to infection, and frequent complications, including scarring and functional impairment, present significant clinical challenges. Severe tissue damage disrupts the skin barrier, leads to fluid loss, and increases the risk of infection, reducing quality of life and increasing healthcare costs [1,2]. Standard treatments, such as basic dressings, creams, and antibiotics, often have limited effect, need frequent changes, and may not fully support healing [2,3]. These challenges highlight the need for new, multifunctional, and biocompatible wound care systems.

Modern wound management relies on the concept of “moist wound healing.” This approach accelerates epithelialization, reduces inflammation and pain, and minimises scar formation [4,5]. Maintaining optimal moisture prevents dehydration and helps absorb exudate, as shown by hydrogel-based dressings [6,7]. The physicochemical properties of wound dressings, such as swelling capacity, moisture retention, and mechanical flexibility, are critical to their clinical performance and therapeutic effect [8,9].

In addition to maintaining a favourable wound environment, controlling oxidative stress is a key factor in promoting tissue regeneration. Burn and chronic wounds are characterised by excessive production of reactive oxygen species, which can prolong inflammation, damage cellular components, and impair healing processes [10,11]. Therefore, the incorporation of antioxidant bioactive compounds into wound dressings has emerged as a promising strategy to mitigate oxidative damage and enhance tissue repair [12]. Plant-derived extracts have been extensively investigated for wound management because they contain a wide range of bioactive constituents, including phenolic compounds (particularly flavonoids), terpenoids, and carotenoids, which are associated with antioxidant, antimicrobial, anti-inflammatory, and tissue-repair-supporting effects [13,14]. For instance, *Calendula officinalis* (marigold) promotes tissue regeneration and reduces inflammation [15], while *Matricaria chamomilla* (chamomile) demonstrates antioxidant and antimicrobial activities [16,17] that support wound healing. *Plantago major* (greater plantain) contains polysaccharides and phenolic compounds that stimulate fibroblast proliferation and angiogenesis [18], whereas *Hippophae rhamnoides* provides carotenoids and essential fatty acids that enhance epithelialization [19]. Additionally, *Hypericum perforatum* has been reported to accelerate wound closure and reduce microbial colonisation due to its flavonoid and hyperforin content [20]. The selection of these species was based on their complementary phytochemical profiles and traditional topical use for skin-related conditions, aiming to combine hydrophilic and lipophilic bioactive constituents within a single emulgel system. Although synergistic interactions among the extracts were not directly investigated in this study, their association was intended to provide functional complementarity through antioxidant, soothing, epithelial-supporting, and skin-protective effects.

In recent years, advanced wound dressings have focused on hydrogel-based systems that maintain hydration, absorb exudate, and deliver therapeutic agents in a controlled manner [21]. Hydrogels are attractive for their high water content, biocompatibility, and ability to conform to wound surfaces. Among natural polymers, sodium alginate—a polysaccharide from brown algae—has been widely used for its excellent gel-forming properties with divalent cations, its biodegradability, and its capacity to create a moist, protective environment that supports epithelialization and tissue regeneration [22,23]. Alginate matrices can also be engineered to modulate mechanical properties, porosity, and release kinetics of bioactive compounds.

Recent studies have highlighted the potential of ionically crosslinked alginate hydrogels with plant-derived extracts for skin regeneration and wound healing [24,25]. These systems benefit from alginate’s biocompatibility and its role in exudate management, while delivering phytochemicals with antioxidant, anti-inflammatory, and antimicrobial effects. Combining alginate matrices with natural extracts supports tissue repair and enhances wound dressing performance. Yet, most formulations have used single extracts or simple hydrogels. In contrast, our main innovation is the integration of both hydrophilic and lipophilic botanical extracts, from multiple complementary plant species, with hyaluronic acid into a printable alginate-based emulgel. This blend is shaped into structurally defined patches using 3D printing after Ca^2+^ crosslinking. Our approach combines a multicomponent phytotherapeutic formulation with extrusion-based 3D printing and post-printing ionic crosslinking, yielding stable, multifunctional emulgel patches that support moisture balance, antioxidant protection, and skin regeneration. Thus, our work advances the field by combining diverse bioactive botanicals and polymers in a printable, 3D-structured system for potential application in burn wound care.

In this context, emulgel-based systems have recently emerged as promising multifunctional biomaterials that combine the hydrophilic characteristics of hydrogels with the ability to incorporate lipophilic therapeutic agents [26,27]. Such systems enable the simultaneous delivery of both hydrophilic and hydrophobic plant-derived compounds, enhancing their therapeutic potential. The integration of these advanced formulations with three-dimensional bioprinting technologies further expands their applicability, allowing precise control over dressing geometry, porosity, and spatial distribution of active ingredients. Three-dimensionally bioprinted wound dressings can therefore provide personalised, structurally tailored solutions with improved mechanical performance and controlled bioactive release.

The advent of 3D printing has opened new avenues for fabricating personalised, structurally precise wound dressings. Three-dimensional-printed patches enable control over geometry, porosity, and the distribution of the active ingredient within the matrix. This enables uniform release profiles and enhanced tissue integration. By adding plant-derived extracts to alginate-based bioinks, multifunctional wound dressings can be designed to provide protection, hydration, and biological stimulation of healing. In this study, the patches had a simple square geometry. Extrusion-based 3D printing was chosen for its accurate and reproducible control of patch dimensions, thickness, layer organisation, and material deposition. Compared with conventional casting, additive manufacturing improves batch reproducibility and offers the potential for customisation to wound size, shape, and therapeutic needs. This work is an initial step toward developing personalised phytotherapeutic wound dressings using additive manufacturing.

Considering these advances, the present study aims to develop and physicochemically characterise a 3D-printed, alginate-based emulgel enriched with selected plant extracts (*Calendula officinalis*, *Matricaria chamomilla*, *Plantago major*, *Hippophae rhamnoides*, and *Hypericum perforatum*). The research focuses on evaluating its structural integrity, swelling behaviour, moisture balance, mechanical flexibility, and antioxidant activity, as well as their suitability as biocompatible wound dressings, providing a basis for further investigation of their therapeutic potential in the management of burns and chronic wounds.

Despite the growing interest in alginate-based wound dressings and plant-derived therapeutics, studies focused on the development of multifunctional 3D-printed alginate emulgel patches incorporating both hydrophilic and lipophilic plant extracts within a single crosslinked delivery platform remain limited. Moreover, comprehensive investigations into the transition from emulgel systems to stabilised crosslinked patch matrices, including their physicochemical, pharmacotechnical, and biocompatibility characteristics, are still lacking. In this context, the aim of the present study was to design and characterise novel 3D-printed alginate emulgel patches intended for potential burn wound applications. The research focused on evaluating the suitability of the alginate-based emulgel for extrusion-based 3D printing and Ca^2+^-induced crosslinking, as well as on assessing the resulting patches in terms of physicochemical properties, structural organisation, flexibility, swelling behaviour, moisture-related parameters, antioxidant potential, in vitro release profile, and preliminary biocompatibility using HaCaT keratinocyte cells.

## 2. Results and Discussion

The present study focused on the development and comprehensive characterisation of alginate-based emulgel formulations, as well as their corresponding 3D-printed patches enriched with plant-derived bioactive compounds. The results are discussed in relation to the physicochemical properties of the formulations and their phytochemical composition, antioxidant activity, and biological compatibility, with particular emphasis on their potential application in burn wound management.

### 2.1. Phytochemical Characterisation of Plant Extracts

The total polyphenol content (TPC) of the hydroglyceric extracts was highest in Calendula officinalis (0.824 ± 0.028 mg GAE/mL), followed by *Plantago major* (0.715 ± 0.021 mg GAE/mL) and *Matricaria chamomilla* (0.671 ± 0.019 mg GAE/mL). These results confirm that all extracts are significant sources of phenolics, with *Calendula officinalis* being especially rich. Their integration into the emulgel matrix is anticipated to enhance the biological performance of the formulations, given their relevance to wound healing.

To further characterise the phytochemical profile of the selected plant extracts, preliminary HPLC fingerprint analyses were performed for all hydroglyceric extracts included in the formulation. The resulting chromatographic profiles, together with the major identified constituents, are provided in the Supplementary Materials (Figures S1–S3), offering additional information on their phytochemical composition.

Oily extracts were incorporated to provide complementary lipophilic bioactive constituents. The *Hippophae rhamnoides* oil extract contained 4.035 mg/100 g total carotenoids (as β-carotene equivalents), suggesting antioxidant molecules with potential for skin regeneration [28]. These may contribute to protective and therapeutic properties in burn wound applications. HPLC-UV analyses confirmed the presence of hypericin-related compounds in *Hypericum perforatum* and characteristic naphthodianthrone derivatives, supporting phytochemical characterisation (Supplementary Materials, Figures S4 and S5).

The results demonstrate the efficient recovery of hydrophilic and lipophilic bioactive constituents, supporting their combined functional contribution and warranting further physicochemical evaluation of the alginate-based emulgel.

### 2.2. Stability and Physicochemical Characterisation of Alginate-Based Emulgel

The developed emulgel was designed as a printable matrix for the fabrication of 3D-printed wound dressings and as a carrier for plant-derived bioactive compounds. Its physicochemical properties were evaluated to assess its suitability for topical application and processing.

#### 2.2.1. Organoleptic Properties

The emulgel exhibited a homogeneous semi-solid appearance, with a light yellow to beige colour attributed to the incorporated plant extracts and oily components. No visible phase separation, aggregates, or air bubbles were observed. The formulation showed a smooth texture and a mild herbal odour, indicating suitable organoleptic characteristics for topical application and subsequent 3D printing.

#### 2.2.2. pH Measurement

The pH of the emulgel remained stable over the 30-day storage period, ranging from 6.02 ± 0.02 to 6.14 ± 0.12 (Figure S2, from Supplementary Materials). These values fall within the physiological skin pH range, supporting the formulation’s suitability for topical application and indicating good chemical stability during storage at 20 °C.

#### 2.2.3. Rheological Behaviour

**Figure 1:**
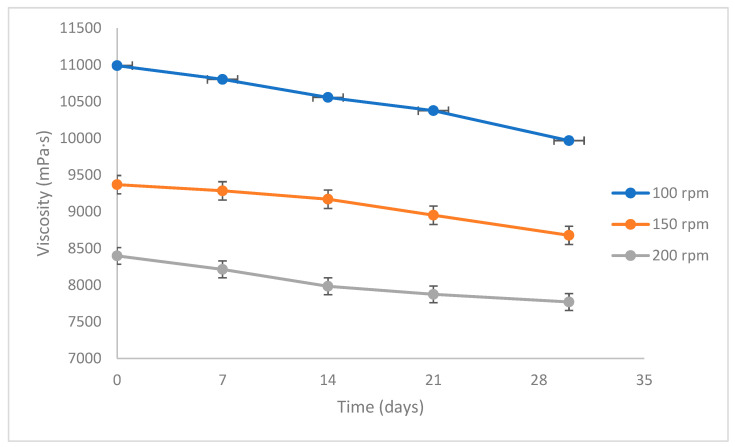
Variation in viscosity of the emulgel at different shear rates (100, 150 and 200 rpm) over 30 days of storage. Data are expressed as mean ± SD.

The viscosity of the emulgel decreased with increasing shear rate, as shown by measurements performed at 100, 150, and 200 rpm (Figure 1). At all time points, the highest viscosity values were recorded at 100 rpm, whereas the lowest were observed at 200 rpm, confirming a non-Newtonian, shear-thinning behaviour characteristic of alginate-based hydrogel and emulgel systems. A slight decrease in viscosity was observed during the 30-day storage period; for example, at 100 rpm, viscosity decreased from 10,990 mPa·s at T0 to 9968 mPa·s at T30, indicating only a minor reduction in structural consistency.

This rheological profile is advantageous for both topical applications and extrusion-based 3D printing, as it promotes easy spreading during application, enables smooth flow through the nozzle, and facilitates structural recovery after deposition. These properties contribute to high shape fidelity after printing and support the suitability of the emulgel as a printable matrix for the fabrication of 3D-printed wound dressings [29,30,31].

#### 2.2.4. Yield Stress Analysis

In addition to the shear-thinning behaviour observed during viscosity measurements, the yield stress of the emulgel was evaluated using the Casson rheological model in order to assess its resistance to flow under low applied stresses and its suitability for extrusion-based 3D printing. Yield stress is the minimum stress required to disrupt the internal structure of a semisolid material and initiate flow, and is recognised as one of the key rheological parameters governing the printability, extrusion behaviour, and shape fidelity of hydrogel- and emulgel-based bioinks intended for biomedical applications [31,32,33].

The linearised Casson plot (Figure S7, Supplementary Materials) showed excellent agreement with the experimental data, yielding a coefficient of determination (R^2^) of 0.9795, indicating that the Casson model adequately describes the rheological behaviour of the developed formulation. The corresponding regression analysis resulted in a Casson yield stress (τ_0_) of 0.324 Pa and a Casson plastic viscosity (ηc) of 3.73 cP·s. The experimental shear rate and shear stress data used for the model fitting, together with the corresponding linearization parameters, are presented in Table S1 of the Supplementary Material.

The existence of a measurable yield stress demonstrates that the emulgel behaves as a structured viscoelastic material capable of maintaining its shape under low-applied-stress conditions while flowing once the applied stress exceeds the critical threshold. Below the yield stress, the formulation behaves predominantly as a viscoelastic solid, maintaining its structural integrity and resisting deformation. Above this threshold, the internal network progressively breaks down, allowing material flow and extrusion through the printing nozzle. Similar rheological behaviour has been reported for printable alginate-based hydrogels and bioinks, where the presence of yield stress contributes significantly to dimensional stability, filament formation, and post-printing structural integrity [31,32,33].

From a practical perspective, the combination of shear-thinning behaviour and measurable yield stress is highly advantageous for extrusion-based 3D printing. The decrease in viscosity under shear facilitates smooth passage of the material through the printing nozzle, while the yield stress promotes rapid structural recovery and shape retention immediately after deposition. These properties are considered essential for achieving high printing fidelity and preventing filament collapse or excessive spreading of the printed constructs [31,32]. Consequently, the developed emulgel exhibits rheological characteristics compatible with bioink-like materials and supports its suitability as a printable matrix for the fabrication of alginate-based wound dressing patches.

#### 2.2.5. Structural Recovery After Extrusion

Structural recovery is a critical rheological parameter in extrusion-based systems because it reflects a material’s ability to rebuild its internal structure after experiencing high shear stresses. Rapid viscosity recovery contributes to shape fidelity and structural stability after deposition. To further evaluate the post-extrusion behaviour of the emulgel, a simplified three-step thixotropic recovery test was performed to assess its structural rebuilding capacity following exposure to high-shear conditions. The initial viscosity measured at 10 rpm was 11,486.7 ± 11.5 mPa·s. Upon the application of high shear (100 rpm, 300 s), the viscosity decreased to 6751.7 ± 206.3 mPa·s, corresponding to a structural retention of 58.8% and a viscosity loss of 41.2%, indicating partial disruption of the internal network under extrusion-like conditions. Following shear cessation and restoration of low-shear conditions (10 rpm), the emulgel retained a high proportion of its initial viscosity, reaching 10,003.3 ± 5.8 mPa·s immediately after the high-shear step, corresponding to 87.10% of the initial viscosity. During the subsequent recovery period, viscosity remained relatively stable, with viscosity recovery ranging from 85.1% to 87.1%, indicating effective preservation and re-establishment of the internal network after shear-induced disruption (Table S2, Supplementary Materials).

Overall, approximately 63.8% of the viscosity lost during high-shear treatment was recovered during the subsequent recovery phase, highlighting the ability of the formulation to rebuild its internal network after shear-induced disruption. The combination of pronounced shear-thinning behaviour, moderate structural disruption under shear, and efficient post-shear recovery suggests that the emulgel possesses rheological characteristics favourable for extrusion-based processing, contributing to structural stability and shape retention after deposition.

#### 2.2.6. Spreadability

**Figure 2:**
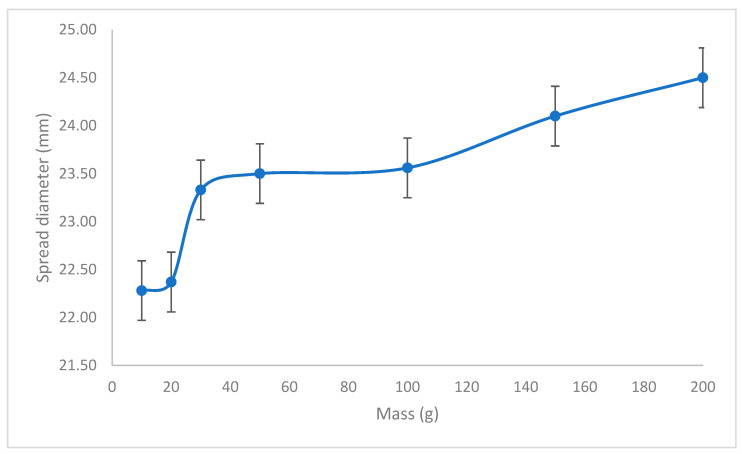
Spreadability of the emulgel as a function of applied weight. Data are expressed as mean ± standard deviation (n = 3).

The spreadability of the emulgel expressed as spreading diameter increased with the applied mass, ranging from 22.28 ± 0.01 mm at 10 g to 24.50 ± 0.03 mm at 200 g, as shown in Figure 2. The gradual increase in spreadability indicates improved deformability under applied stress, although at higher loads (≥100 g) the effect became less pronounced, suggesting a tendency toward a plateau. This behavior is characteristic of semisolid systems and reflects an appropriate balance between flowability and structural integrity, supporting easy skin application while maintaining adequate consistency.

#### 2.2.7. Physical Stability

The physical stability of the emulgel was evaluated under different stress conditions, including temperature variation, centrifugation, and freeze–thaw cycles. The formulation remained physically stable at 4 ± 2 °C and 25 ± 2 °C, with no noticeable changes in appearance, colour, odour, or homogeneity (Table S3, Supplementary Materials). At 37 ± 2 °C, slight changes in consistency and texture were observed, accompanied by a minor increase in spreadability; however, no phase separation occurred, indicating acceptable stability under elevated temperature conditions [34,35].

Centrifugation confirmed the physical integrity of the emulgel, as no phase separation was detected. In addition, the creaming index was approximately 1%, indicating minimal instability and a well-dispersed internal phase, consistent with stable emulsion-based systems [34]. The freeze–thaw study further demonstrated the robustness of the formulation, as no significant phase separation occurred after three cycles between −40 °C and +40 °C, although a slight decrease in viscosity was observed (Table S4, Supplementary Materials). These findings indicate that the emulgel maintains its structural integrity under thermal stress conditions, in agreement with pharmaceutical stability assessment guidelines [36,37].

Overall, the results indicate good physical stability and support the suitability of the emulgel as a printable matrix for 3D-printed patch systems, consistent with the behavior previously reported for hydrogel-based materials [38,39].

#### 2.2.8. Total Polyphenol Content

The total polyphenol content (TPC) was determined to evaluate the incorporation of phenolic bioactive compounds within the emulgel matrix. The alginate-based emulgel exhibited a TPC value of 0.2146 ± 0.014 mg GAE/g, confirming the presence of phenolic constituents derived from the incorporated hydroglyceric plant extracts. This result indicates that the formulation process allowed the retention of polyphenolic compounds within the emulgel system, supporting its functional potential as a bioactive printable matrix.

### 2.3. Fabrication of Emulgel-Based Patches by 3D Printing

**Figure 3:**
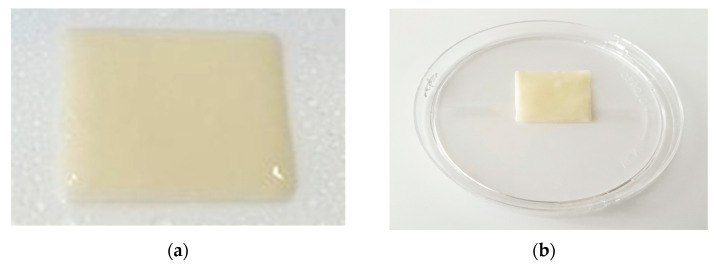
Representative alginate-based 3D-printed patch showing regular geometry and a smooth surface: (**a**) immediately after 3D printing; (**b**) after crosslinking.

The 3D printing process enabled the fabrication of uniform and reproductible emulgel-based patches while preserving the structural integrity of the initial matrix and allowing controlled geometry and composition, which are essential for wound dressing applications. The obtained patches exhibited a well-defined square geometry and a homogeneous structure, with a whitish-yellow appearance and soft consistency (Figure 3a).

Recent studies by Briganti et al. [24], and Zhu et al. [25] have reported alginate-based hydrogels loaded with plant-derived extracts as promising platforms for skin regeneration and wound healing applications due to their favorable biocompatibility, antioxidant potential, and moisture-retention capacity. However, most of these systems were developed as conventional hydrogels or films containing a single botanical extract. In contrast, the present formulation was designed as a printable emulgel incorporating both hydrophilic and lipophilic plant extracts, allowing extrusion-based fabrication of structurally defined patches prior to Ca^2+^-induced stabilization.

Following ionic crosslinking with a 2% (*w*/*v*) calcium chloride solution at room temperature, the patches became slightly more compact while preserving their overall morphology, indicating successful ionic crosslinking of the alginate matrix (Figure 3b). No visible deformation, fragmentation, or dimensional changes were observed during handling or after storage, confirming the integrity of the developed patches.

The selected 3D printing parameters ensured stable extrusion and accurate filament deposition, resulting in uniform patch architectures. The absence of defects, such as irregular filament formation or collapse during printing, indicates that the applied conditions were suitable for maintaining the rheological behavior of the emulgel and ensuring the quality of the final constructs. Since the printing, crosslinking, and drying steps may influence key properties such as mechanical strength, swelling behavior, and moisture management, further pharmacotechnical and physicochemical characterization was performed to further assess their suitability for burn wound applications.

### 2.4. Pharmacotechnical and Physicochemical Characterisation of 3D-Printed Patches

The printed patches were evaluated in terms of uniformity, mechanical performance, moisture-related behavior, and structural characteristics, all of which are essential for their function as wound dressings.

#### 2.4.1. Weight Uniformity

The weight uniformity analysis showed a mean patch weight of 0.7436 ± 0.0187 g, indicating the good consistency of the fabrication process.

#### 2.4.2. Dimensional Analysis

Dimensional analysis confirmed the square geometry of the patches, with a mean side length of 22.88 ± 0.14 mm and a thickness of 1.47 ± 0.23 mm, demonstrating good dimensional reproducibility after 3D printing.

#### 2.4.3. Folding Endurance

The folding endurance exceeded 300 folds for all samples (n = 3); therefore, no standard deviation could be calculated. These values are consistent with those reported for similar hydrogel-based or polymeric patches (approximately 340–490 folds), indicating excellent flexibility and mechanical resistance [40,41]. Such behavior suggests that the patches can withstand repeated mechanical stress during handling and application without structural damage.

#### 2.4.4. Loss on Drying

The mean moisture content of the 3D-printed patches was 90.02 ± 1.10%, indicating a high water-retention capacity. All measurements were performed in triplicate, and the low variability among replicates suggests good formulation uniformity and process reproducibility.

This behavior can be attributed to the hydrophilic composition of the matrix, particularly the presence of sodium alginate, hyaluronic acid, and glycerin. Such high moisture content is characteristic of hydrogel-based systems and may contribute to maintaining a moist environment at the application site, which is beneficial for skin hydration and wound healing [42,43]. In addition, retained water may improve the flexibility of the patches and facilitate their adaptation to irregular surfaces.

#### 2.4.5. Surface pH

The surface pH of the 3D-printed patches was evaluated in distilled water and phosphate-buffered solution (PBS, pH 7.4) to assess their behavior under different hydrated conditions. The results are presented in Table 1 and Table 2. In distilled water, the patches showed slightly acidic pH values, ranging from 6.28 to 6.44, which are close to the physiological skin pH, showing good suitability for topical application. In PBS, the surface pH shifted toward neutral values, ranging from 7.29 to 7.37, reflecting the buffering capacity of the medium.

The low standard deviation values and the minimal variation observed over time indicate good measurement reproducibility and pH stability upon hydration. Moreover, the similarity between the two batches confirms the consistency of the formulation and the reproducibility of the 3D-printing process. Overall, these results suggest that the developed patches maintain appropriate surface pH characteristics under different environmental conditions and are unlikely to induce irritation during topical application.

These findings are consistent with previous reports on transdermal patch systems, where surface pH values within the physiological range are associated with good skin compatibility and reduced risk of irritation. The stability of pH under hydrated conditions further supports the suitability of the developed patches for biomedical applications. Moreover, the obtained values are in agreement with literature data and relevant standards [44,45], which indicate that patches exhibiting surface pH within the physiological range are generally well tolerated.

#### 2.4.6. Swelling Capacity

The swelling capacity and water vapour transmission rate (WVTR) were assessed to determine the ability of the patches to manage wound exudate and maintain an appropriate moisture balance at the wound interface. A swelling capacity of 58.24% was obtained, indicating effective fluid uptake, which may be attributed to the hydrophilic nature of the alginate–hyaluronic acid matrix and its porous microstructure. This property is particularly relevant for burn wound management, as it promotes exudate absorption while preserving a moist environment favourable for tissue regeneration [38]. In addition, the patches retained their structural integrity upon hydration, further supporting their suitability as wound dressings.

#### 2.4.7. Water Vapour Transmission Rate (WVTR)

The WVTR value of 1270 ± 93 g/m^2^/24 h falls within the range reported for semi-occlusive dressings, indicating adequate permeability for controlled water vapour exchange [42,43]. Such behaviour is essential to prevent excessive tissue dehydration while avoiding fluid accumulation at the wound site. Taken together, these findings indicate that the developed patches are capable of simultaneously absorbing wound exudate and regulating moisture, supporting their multifunctional character and potential utility in burn wound management.

#### 2.4.8. Total Polyphenol Content

The total polyphenol content (TPC) of the 3D-printed patches was determined to evaluate the retention of bioactive compounds after the gel-to-patch transformation. A TPC value of 0.2878 ± 0.016 mg GAE/g hydrated crosslinked patch was obtained, corresponding to approximately 2.878 mg GAE/g dry patch equivalent, considering the 90% loss on drying. This value was higher than that measured for the corresponding emulgel formulation (0.2146 mg GAE/g), most likely due to changes in water content and matrix densification during Ca^2+^-induced crosslinking, resulting in a relative concentration of phenolic compounds within the final patch matrix. Matrix-dependent extraction behaviour may also contribute to this difference, since phenolic compounds in the semisolid emulgel may be partially retained within the polymeric network or distributed between phases, thereby limiting their accessibility during extraction [46,47]. By contrast, the consolidated patch matrix may facilitate more efficient recovery of these compounds. Therefore, the higher TPC observed in the patches should be interpreted primarily as a mass-normalisation effect and relative concentration rather than as an increase in the absolute amount of polyphenols. Overall, these results indicate that the printing and crosslinking processes did not compromise the phytochemical integrity of the formulation and support the retention of plant-derived antioxidant constituents in the final patch system [48].

### 2.5. Structural Characterisation of the Emulgel and 3D-Printed Patches

Structural characterization was performed to investigate the morphology and molecular interactions of the emulgel and the corresponding 3D-printed patches following the printing and crosslinking process.

#### 2.5.1. Scanning Electron Microscopy (SEM) Analysis

**Figure 4:**
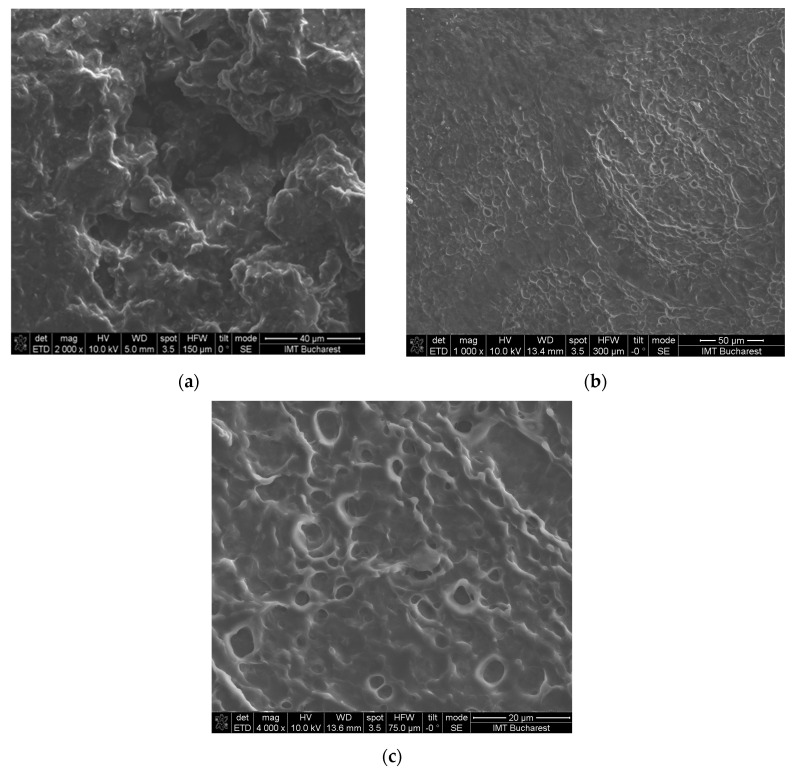
SEM micrographs of the alginate-based emulgel and Ca^2+^-crosslinked alginate patch: (**a**) emulgel; (**b**) crosslinked patch at 1000× magnification; (**c**) crosslinked patch at 4000× magnification.

The SEM micrographs presented in Figure 4 illustrate the structural transition from the alginate-based emulgel to the Ca^2+^-crosslinked alginate patch, highlighting significant differences in morphology, organization, and phase distribution.

As shown in Figure 4a, the emulgel exhibited a heterogeneous and irregular morphology, reflecting the coexistence of the hydrophilic alginate matrix with dispersed lipid domains and plant-derived components. Similar heterogeneous structures have previously been reported for emulgel systems, where multiple phases coexist within a semisolid matrix and are stabilized through weak intermolecular interactions such as hydrogen bonding and van der Waals forces [49,50].

In contrast, the SEM image of the Ca^2+^-crosslinked alginate patch (Figure 4b) revealed a more compact, continuous, and homogeneous surface morphology. This structural organization is consistent with the formation of a three-dimensional ionically crosslinked network resulting from interactions between Ca^2+^ ions and alginate carboxylate groups. Similar morphological changes have been reported for calcium alginate systems, where ionic crosslinking increases matrix cohesion, packing density, and structural stability [29,51,52]. Higher-magnification SEM imaging (Figure 4c) revealed the presence of rounded microdomains and small surface depressions distributed throughout the crosslinked matrix. These features are likely associated with entrapped lipid-rich domains originating from the emulgel formulation and further support the heterogeneous composite nature of the crosslinked patch.

The surface of the crosslinked patch exhibited an interconnected architecture together with uniformly distributed rounded microdomains. These features are likely associated with lipid-rich domains originating from the emulgel formulation and physically entrapped within the polymeric network during the crosslinking process. Similar spherical structures have been described in emulgel-derived hydrogel systems, where dispersed oil droplets remain immobilized within the crosslinked matrix after gelation [49,53,54,55].

Overall, the observed morphological modifications support the transformation of the emulgel into a structurally stabilized alginate patch and are consistent with the formation of a homogeneous calcium-crosslinked network.

#### 2.5.2. Scanning Electron Microscopy Coupled with Energy-Dispersive X-Ray Spectroscopy (SEM–EDX)

**Figure 5:**
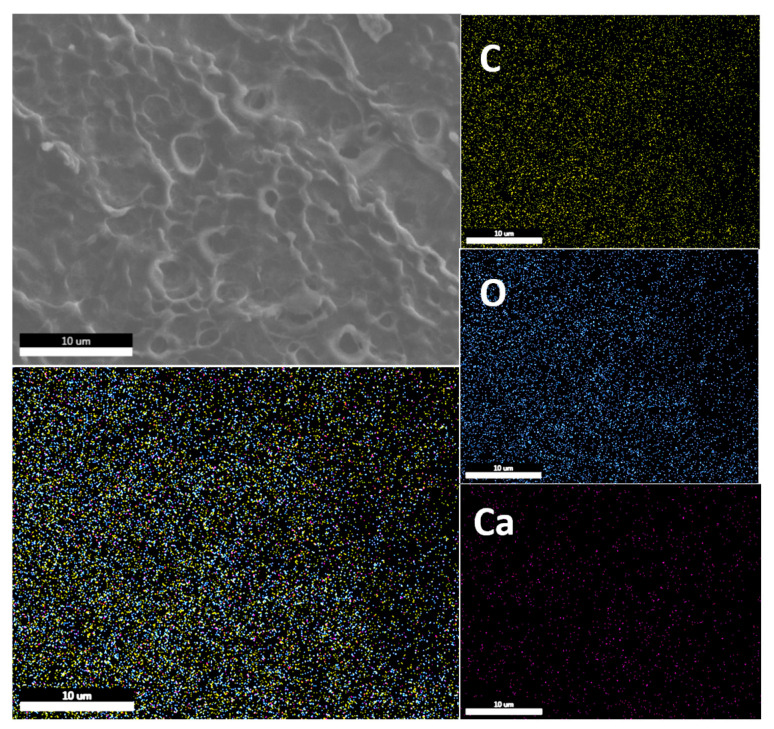

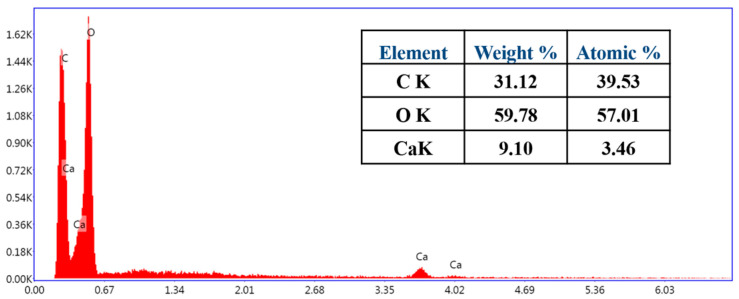
SEM micrograph and corresponding EDX elemental mapping of the Ca^2+^-crosslinked alginate patch showing the distribution of carbon (C), oxygen (O), and calcium (Ca), together with the EDX spectrum and elemental composition table. The homogeneous distribution of calcium confirms the successful ionic crosslinking of the alginate matrix.

To further investigate the incorporation and distribution of calcium within the crosslinked alginate matrix, SEM-EDX analysis was performed (Figure 5). The EDX spectrum confirmed the presence of calcium in the crosslinked patch, with an atomic percentage of 3.46 at.% and a weight percentage of 9.10 wt.%, providing direct evidence of successful ionic crosslinking between Ca^2+^ ions and the carboxylate groups of alginates. Carbon and oxygen were identified as the predominant elements, accounting for 39.53 at.% and 57.01 at.%, respectively, consistent with the polysaccharide-based composition of the matrix. Elemental mapping further demonstrated a homogeneous distribution of calcium throughout the analyzed surface, indicating uniform penetration of the crosslinking solution and the formation of a relatively homogeneous ionic network. The absence of localized calcium-rich regions suggests that the crosslinking process occurred uniformly within the analyzed area. These findings support the morphological observations obtained by SEM and provide complementary evidence for the successful formation of the Ca^2+^-crosslinked alginate structure.

The combined SEM and SEM-EDX results confirm that calcium ions were effectively incorporated into the polymer network, contributing to the densification and structural organization observed after crosslinking.

#### 2.5.3. Mechanical Characterisation by AFM Nanoindentation

Mechanical characterization of the Ca^2+^-crosslinked alginate patch by AFM nanoindentation yielded an average Young modulus of 46.2 kPa. This value indicates the formation of a soft and compliant hydrogel network, which is advantageous for wound dressing applications, allowing adaptation to the wound surface while maintaining structural integrity. Representative force–distance curves used for Young’s modulus determination are presented in Figure S8 (Supplementary Materials). Similar elastic moduli in the kPa range have been reported for soft calcium-crosslinked alginate hydrogels and other hydrogel-based tissue engineering scaffolds characterized by AFM indentation techniques [56].

#### 2.5.4. Dynamic Light Scattering (DLS) Analysis

**Figure 6:**
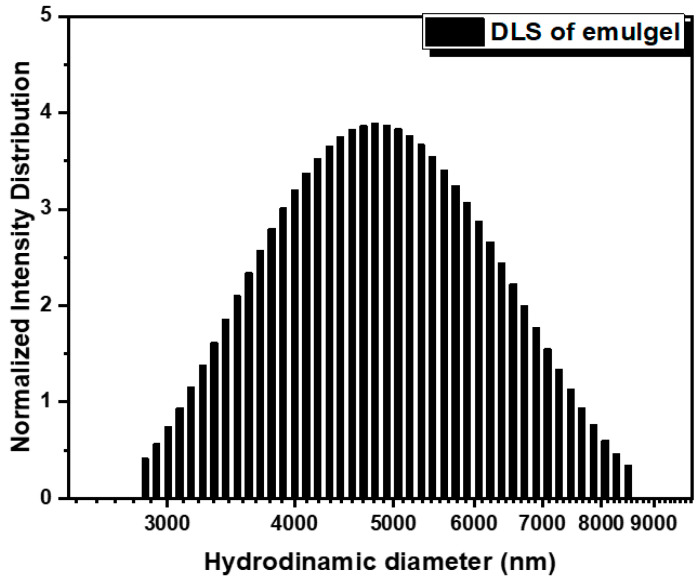
Dynamic light scattering (DLS) analysis showing the hydrodynamic diameter distribution of the emulgel droplets/aggregates in the aqueous phase.

The DLS analysis of the emulgel revealed a broad hydrodynamic diameter distribution centered in the micrometric range, indicating the presence of dispersed droplets and/or aggregates within the aqueous phase (Figure 6).

This polydisperse profile is typical of emulgel systems, in which lipid droplets, polymer chains, and plant-derived constituents coexist [49]. The micrometric size range supports the formation of emulsion-type structures rather than nanoscale dispersions, while also correlating with the heterogeneous morphology observed by SEM.

Due to the complex nature of the emulgel system, which contains polymeric chains, lipid droplets, plant-derived constituents, and associated domains, the DLS measurements should be interpreted as providing an apparent hydrodynamic size distribution rather than an absolute particle size determination. Because DLS is highly sensitive to larger particles and aggregates, broad distributions are commonly expected in viscous semisolid systems such as emulgels. Therefore, the obtained micrometric distribution most likely reflects the coexistence of dispersed droplets, hydrated polymer regions, and associated structures within the aqueous phase.

Although the calculated PDI value was not considered physically reliable due to the semisolid and highly viscous nature of the alginate emulgel, the monomodal distribution profile suggested the absence of major secondary populations or extensive aggregation phenomena. This behavior may indicate a relatively uniform dispersion state of the dominant dispersed domains within the formulation, supporting its apparent physicochemical stability.

The zeta potential value of approximately −20.47 mV indicates moderate colloidal stability. The negative surface charge may be attributed to ionized carboxylate groups (–COO^−^) from alginate, which contribute to electrostatic repulsion between dispersed domains. However, the magnitude of this value suggests limited electrostatic stabilization, indicating that the stability of the emulgel is likely governed by a combination of electrostatic and steric effects [57]. The original DLS/zeta potential correlograms and measurement parameters are included in the Supplementary Materials (Figure S9A,B).

Overall, DLS and zeta potential analysis provide information on the initial dispersion state of the emulgel prior to crosslinking. Following Ca^2+^-induced crosslinking, alginate chains form a three-dimensional ionic network through interactions with carboxylate groups, leading to the formation of a solid patch. Therefore, the DLS results should be interpreted as representative of the pre-gelation state, whereas the cross-linked patches correspond to a structurally stabilized system that is no longer suitable for DLS analysis.

#### 2.5.5. Fourier Transform Infrared Spectroscopy (FTIR) Analysis

**Figure 7:**
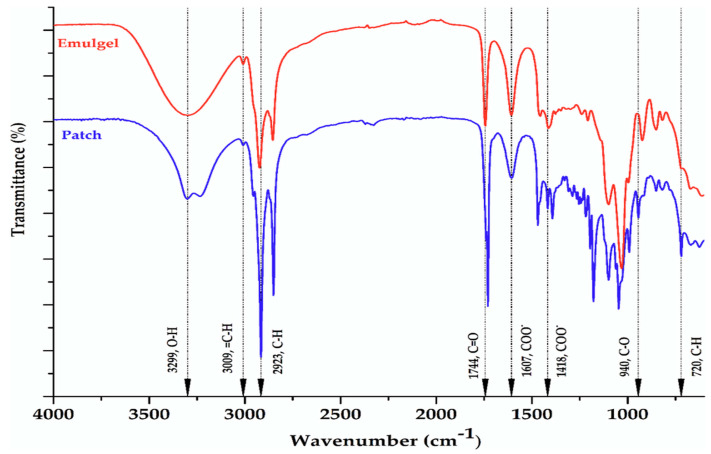
ATR-FTIR spectra of the emulgel and the corresponding alginate-based patch.

Figure 6 presents the ATR-FTIR spectra of the emulgel before and after crosslinking, while the main band assignments are summarised in Table S5 from the Supplementary Materials. The emulgel was a complex formulation containing sodium alginate, hyaluronic acid, glycerol, sea buckthorn oil, Tween 80, vitamin E, and other minor constituents. As expected, the absorption bands of components present at low concentrations were partially masked by the dominant signals of the major formulation components, as shown in Figure 7.

The ATR-FTIR spectrum of the emulgel showed absorption bands mainly associated with the alginate, glycerol, polysorbate, and lipid phases, without the appearance of new bands indicative of covalent bond formation. This suggests that the system is primarily stabilized by physical interactions among the main constituents. The broad band observed in the 4000–3100 cm^−1^ region was attributed to O–H stretching vibrations from glycerol, polysorbate, alginate, and phenolic compounds, indicating the presence of an extended hydrogen-bonding network that contributes to the physical stabilization of the emulgel.

The incorporation of sea buckthorn oil into the polysaccharide matrix was supported by absorption bands assigned to C–H vibrational modes of aliphatic chains and unsaturated groups, including cis-olefinic conformations. The characteristic band at 1744 cm^−1^, assigned to ester carbonyl (C=O) stretching vibrations of triglycerides, further confirmed the presence of lipid constituents derived from sea buckthorn oil [58,59,60]. Moreover, the intensity ratio between the bands at 3009 and 2923 cm^−1^, which can be used as an indicator of unsaturation degree, decreased from 0.44 in the raw sea buckthorn oil to 0.25 in the emulgel, suggesting dilution and partial masking of lipid signals within the polymeric matrix.

The bands observed at 1607 and 1415 cm^−1^ were assigned to the asymmetric and symmetric stretching vibrations of COO^−^ groups from sodium alginate, respectively [61,62]. Compared with pure alginate, slight shifts toward higher wavenumbers were observed in the emulgel spectrum, supporting the presence of physical interactions between alginate and the other bioactive constituents. Additional bands associated with aliphatic –CH_2_ and –CH_3_ deformation vibrations were observed at 1457 cm^−1^ and within the 1400–1300 and 1300–1240 cm^−1^ regions, while C–O stretching vibrations appeared in the 1250–990 cm^−1^ interval [63]. The bands at 819 and 718 cm^−1^ were attributed to guluronic and mannuronic acid residues in the alginate structure [61].

The alginate patch corresponded to the CaCl_2_-crosslinked emulgel. As shown in Figure 7, both samples displayed similar FTIR profiles, confirming the preservation of the main formulation components and suggesting that the system is governed predominantly by physical interactions. After CaCl_2_ treatment, slight changes in band intensity and position were observed, indicating matrix reorganization induced by ionic crosslinking.

The broad absorption band in the 4000–3100 cm^−1^ region showed modifications in shape and intensity after crosslinking, suggesting rearrangement of hydrogen-bonding interactions and reduced mobility of hydroxyl groups within the stabilized network. In addition, the decrease in the intensity ratio of the bands at 3009 and 2916 cm^−1^ to 0.091 may indicate changes in the unsaturated lipid fraction, possibly associated with the partial transformation of cis homologues under thermal or oxidative conditions.

The most relevant spectral changes were observed in the carboxylate region, particularly around 1469 and 1415 cm^−1^, where band splitting, shifting, and narrowing indicated electrostatic interactions between Ca^2+^ ions and alginate carboxyl groups [62,64]. These modifications support the formation of interchain ionic bridges and stabilization of the alginate network. The appearance of multiple bands in the 1330–1000 cm^−1^ region, together with the shoulder at 1062 cm^−1^ and the band at 944 cm^−1^ assigned to guluronate residues, further supports the formation of the characteristic calcium alginate “egg-box” structure [61,65].

The preservation of the lipid bioactive phase, including sea buckthorn oil and vitamin E, was supported by the persistence of characteristic absorption bands in the 3000–2800 cm^−1^, 1740 cm^−1^, and 720 cm^−1^ regions. Overall, the transition from emulgel to Ca^2+^-crosslinked patch led to reduced polymer chain mobility and the formation of a more ordered internal network, while maintaining the integrity of the incorporated bioactive components.

### 2.6. Functional and Biological Evaluation

The biological and functional properties of the developed emulgel and 3D-printed patches were further investigated through antioxidant activity, in vitro release, and biocompatibility studies to evaluate their potential applicability in burn wound management.

#### 2.6.1. Antioxidant Activity

The antioxidant activity of the emulgel and 3D-printed patches was evaluated using the ABTS assay and expressed as both IC_50_ values and Trolox-equivalent concentrations. A concentration-dependent response was observed for both systems, with the patches exhibiting higher antioxidant activity than the corresponding emulgel at equivalent concentrations.

The IC_50_ value of the emulgel was estimated at 8.26 mg/mL, whereas the patches showed a lower IC_50_ value of 5.63 mg/mL, indicating stronger radical scavenging activity after conversion into the dried matrix. As expected, Trolox displayed the highest antioxidant activity, with an IC_50_ value of 6.42 µmol/mL and was used as a positive control.

The same trend was observed when the results were expressed as Trolox equivalents. At 4 mg/mL, the patches reached 5.22 µmol/mL Trolox equivalents, compared with 4.23 µmol/mL for the emulgel, with similar differences also observed at 2 and 1 mg/mL. The enhanced antioxidant performance of the patches may be attributed to matrix reorganization following Ca^2+^-induced crosslinking and rinsing, which may influence the extractability and availability of phenolic constituents within the final patch matrix. This interpretation is consistent with the higher total polyphenol content determined for the patches compared with the emulgel.

From a functional perspective, the stronger antioxidant activity of the patch system is relevant for burn wound applications, where oxidative stress may impair tissue regeneration. These results support the potential of the developed patches to provide antioxidant protection at the wound interface and contribute to favorable healing conditions.

#### 2.6.2. In Vitro Polyphenol Release from 3D-Printed Patches

**Figure 8:**
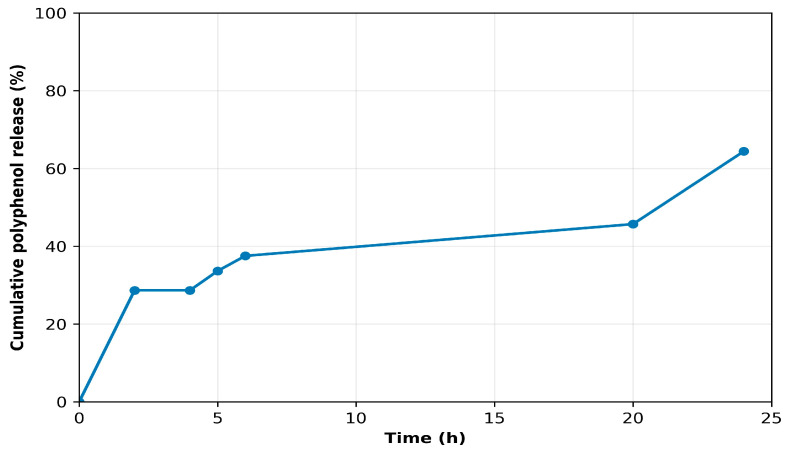
In vitro cumulative release profile of polyphenols from 3D-printed patches over 24 h, expressed as percentage of total polyphenol content, calculated as gallic acid equivalents (GAE).

The in vitro release profile of polyphenols from the 3D-printed patches is presented in Figure 8. Cumulative release data were corrected for the volume removed at each sampling point, as the dissolution medium was not replaced during the experiment. Sink conditions were maintained throughout the study, as the maximum measured concentration remained well below the polyphenol solubility limit in the PBS–ethanol medium. It should be acknowledged that the present release study was performed in PBS (pH 7.4)–ethanol (70:30, *v*/*v*), rather than in simulated wound fluid. The hydroalcoholic medium was selected to maintain sink conditions for the heterogeneous pool of hydrophilic and lipophilic bioactive constituents present in the formulation. However, this medium does not fully reproduce the protein content, ionic composition, and dynamic environment of wound exudate. Therefore, the cumulative release values reported here should be interpreted as formulation-specific in vitro descriptors obtained under standardised conditions rather than as direct predictors of in vivo release behaviour.

The patches exhibited a biphasic release profile, with an initial burst of 28.68% within the first 2 h, likely associated with the rapid diffusion of phenolic compounds located at or near the patch surface. Between 2 and 4 h, the cumulative release remained nearly unchanged, whereas after 4 h it gradually increased, reaching 33.66% at 5 h, 37.54% at 6 h, 45.71% at 20 h, and 64.42% at 24 h. These values corresponded to adjusted amounts of released polyphenols ranging from 3.12 to 7.01 µg GAE. This behaviour is consistent with a time-dependent release process involving diffusion through the hydrated alginate matrix together with possible interactions between polyphenols and the crosslinked polymeric network.

To further investigate the release mechanism, the experimental data were fitted to zero-order, first-order, Higuchi, Korsmeyer–Peppas, and Weibull models (Table S6, Supplementary Materials). The obtained R^2^ values were relatively modest and close to each other, indicating that no single classical kinetic model clearly dominated the release profile. This may be related to the limited number of sampling points, the biphasic release pattern, the apparent plateau between 2 and 4 h, and the heterogeneous nature of the composite alginate-based matrix.

The Korsmeyer–Peppas model, applied to the data points with Mt/M∞ < 0.60, yielded a release exponent of n = 0.221 (R^2^ = 0.866). This value is markedly lower than that generally reported for ideal Fickian diffusion from slab-like systems, indicating that the release behaviour cannot be adequately described by a simple homogeneous diffusion process.

Instead, it most likely reflects the combined influence of several formulation-dependent phenomena, including the initial burst release of surface-accessible polyphenols, the near-plateau observed between 2 and 4 h, restricted mobility of bioactive compounds within the Ca^2+^-crosslinked alginate network, possible reversible interactions between phenolic hydroxyl groups and alginate carboxylate groups, and partitioning of the bioactive constituents between the hydrophilic polymeric phase, lipophilic oil domains, and their interfaces. Therefore, the Korsmeyer–Peppas exponent is best regarded as an apparent empirical parameter describing a heterogeneous multi-domain release system rather than as definitive evidence of a single transport mechanism.

This interpretation is supported by the Weibull shape parameter (b = 0.434) and by the Higuchi model, both of which indicate that diffusion through the hydrated matrix represents one of the main release-controlling processes. The lower fit of the first-order model further suggests that release was not governed solely by the residual concentration of phenolic constituents within the matrix. Similar biphasic release profiles have been reported for 3D-printed alginate-based patches containing plant-derived bioactive compounds, where diffusion-controlled release mechanisms were also observed [66,67,68].

Overall, the patches enabled the time-dependent release of polyphenolic compounds over 24 h, combining an early release phase with subsequent gradual diffusion. This profile may be advantageous for topical applications, where an initial antioxidant contribution followed by prolonged availability of phenolic constituents could support a favorable wound microenvironment. Further studies using extended release periods and simulated wound exudate media are warranted to better define the release mechanism and functional relevance of the system.

#### 2.6.3. Biocompatibility Assessment

HaCaT keratinocytes were used as an in vitro model to evaluate the biocompatibility of the emulgel patches, as these cells are widely employed in studies related to epidermal biology, skin regeneration, and wound healing [69]. Keratinocytes play a key role in re-epithelialization by supporting cell migration, proliferation, and restoration of the epidermal barrier, making the preservation of their viability essential for topical systems intended for wound management [70].

**Figure 9:**
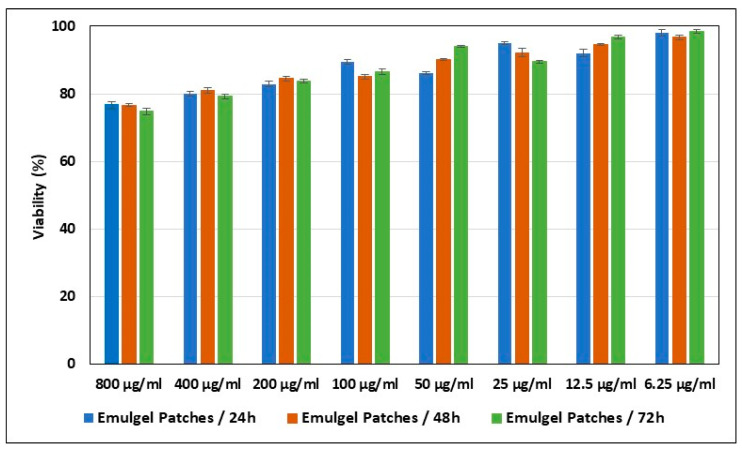
Effect of the developed emulgel patches on the viability of HaCaT human keratinocytes after 24, 48, and 72 h of exposure at concentrations ranging from 6.25 to 800 µg/mL. Untreated cells were considered to have 100% viability. Data are presented as mean ± standard deviation (SD), n = 3.

Cell viability was assessed after exposure to different formulation concentrations (6.25–800 µg/mL) for 24, 48, and 72 h (Figure 9). The results showed a slight concentration- and time-dependent decrease in HaCaT viability. However, at lower concentrations, cell viability remained above 90%, indicating minimal interference with cellular metabolic activity and supporting the cytocompatibility of the patches [71].

At higher concentrations and longer exposure times, particularly after 48–72 h, a moderate reduction in viability was observed. This effect may be related to prolonged interactions between released bioactive compounds, polymeric matrix components, and the cellular microenvironment. Nevertheless, viability values remained within acceptable limits for topical biomaterial-based formulations, suggesting the absence of severe cytotoxic effects. In addition, treated cells preserved their characteristic epithelial-like morphology, further supporting compatibility with epidermal cells.

Overall, the results indicate that the alginate-based emulgel patches exhibit a favorable biocompatibility profile on HaCaT keratinocytes. This behavior may be attributed to the hydrophilic and biocompatible nature of the alginate-based matrix, which can support a hydrated microenvironment while limiting direct cellular stress. These findings support the potential suitability of the patches for topical wound-related applications.

Taken together, the obtained results highlight the complex functional profile of the developed patches. Their multifunctional character arises from the synergistic integration of a biopolymeric matrix with plant-derived bioactive compounds, enabling simultaneous physical protection, fluid management, and biological activity.

Although the 3D-printed alginate emulgel patches showed promising physicochemical, functional, and biocompatibility properties, several limitations should be acknowledged. The release study was performed over 24 h using total polyphenol content as a global marker rather than individual phenolic compounds, and the experiments were conducted under simplified in vitro conditions. Moreover, biocompatibility was evaluated using a keratinocyte monoculture model, which does not fully reproduce the complexity of burn wound tissue. Future studies should include extended release testing, identification and quantification of individual bioactive compounds, evaluation in simulated wound exudate, antimicrobial and anti-inflammatory assays, and advanced biological models, including co-culture systems, ex vivo skin models, and in vivo burn wound studies.

## 3. Conclusions

This study showed that 3D-printed alginate-based emulgel patches with both hydrophilic and lipophilic plant extracts could serve as multifunctional systems for potential burn wound applications. The extracts delivered relevant bioactive constituents—polyphenols, carotenoids, and hypericin-related compounds—which contributed to the formulation’s biological functionality.

The developed emulgel exhibited physicochemical, rheological, and stability characteristics suitable for extrusion-based 3D printing, enabling the fabrication of uniform and reproducible patches. Following Ca^2+^-induced crosslinking, the patches maintained their structural integrity and displayed properties compatible with wound dressing applications, including flexibility, appropriate surface pH, swelling capacity, and adequate water vapour transmission. SEM, SEM–EDX, FTIR, and DLS analyses collectively confirmed the formation of a stabilised calcium-crosslinked alginate network while preserving the main formulation components.

The resulting patches retained their phytochemical functionality. They showed enhanced antioxidant activity compared with the initial emulgel. The patches also exhibited a time-dependent release of phenolic compounds over 24 h, mainly driven by diffusion through the hydrated matrix. In addition, HaCaT keratinocyte viability assays showed a favourable biocompatibility profile. This supports the suitability of the developed system for topical applications.

Taken together, these findings show that the proposed 3D-printed Ca^2+^-crosslinked alginate emulgel patches offer structural stability, moisture-regulating properties, bioactive compound delivery, antioxidant activity, and cytocompatibility. This highlights their potential as advanced bioactive dressings for burn wound care.

The encouraging results reported in this study support further investigation of the developed system. Future work should include extended-release studies in simulated wound exudate, the identification and quantification of individual released phytoconstituents, antimicrobial and anti-inflammatory evaluations, and testing in advanced biological models such as keratinocyte–fibroblast co-cultures, ex vivo skin models, and in vivo burn wound-healing studies. Such investigations will help provide a more comprehensive assessment of the therapeutic performance and clinical applicability of the proposed patch system.

## 4. Materials and Methods

### 4.1. Materials

Flowers of *Calendula officinalis* L. and *Matricaria chamomilla* L. (chamomile), together with fruits of *Hippophae rhamnoides* L. (sea buckthorn), were collected in July 2025 from Dumitrești village, Vrancea County, Romania. Leaves of *Plantago major* L. (greater plantain) and aerial parts of *Hypericum perforatum* L. (St. John’s wort) were harvested in August 2025 from Stoenești village, Argeș County, Romania. The plant species were identified at the Botany Laboratory of the Faculty of Pharmacy, Titu Maiorescu University, according to standard botanical identification procedures.

### 4.2. Chemicals

Sodium hyaluronate, sodium acetate, Folin–Ciocalteu reagent, gallic acid, sodium carbonate, phosphate-buffered saline (PBS, pH 7.4), 2,2′-azinobis(3-ethylbenzothiazoline-6-sulfonic acid) (ABTS), and potassium persulfate were purchased from Sigma-Aldrich Chemie GmbH (Taufkirchen, Germany). Calcium chloride, sodium alginate, polysorbate 80, glycerol, and ethanol were obtained from Merck (Darmstadt, Germany). Olivem^®^ 1000, tocopheryl acetate, sunflower oil, and Cosgard were acquired from Elemental SRL (Oradea, Romania). Purified water was used throughout the experiments.

### 4.3. Preparation and Characterisation of Plant Extracts

Hydroglyceric extracts of *Calendula officinalis*, *Matricaria chamomilla*, and *Plantago major* were prepared by mixing finely powdered dried plant material with a glycerol–water mixture (40:60, *w*/*w*) at a plant material concentration of 10% (*w*/*w*). The mixtures were sonicated for 2 h at room temperature using an ultrasonic bath (Witeg Labortechnik, Wertheim, Germany), filtered through Whatman No. 1 filter paper, and stored at 4 °C until further use.

Oily extracts of *Hippophae rhamnoides* fruits and *Hypericum perforatum* aerial parts were obtained by cold maceration in sunflower oil (1:10, *w*/*w*) for four weeks in the dark at room temperature, with occasional stirring. The resulting extracts were warm-filtered through sterile muslin cloth and stored at low temperature until use.

The total polyphenol content of the hydroglyceric extracts was determined using the Folin–Ciocalteu colorimetric method and expressed as mg gallic acid equivalents per mL extract (mg GAE/mL). The total carotenoid content of the *Hippophae rhamnoides* oil extract was determined spectrophotometrically and expressed as β-carotene equivalents. Furthermore, all plant extracts incorporated into the formulation were characterized by HPLC-UV fingerprint analysis to further evaluate their phytochemical composition. In the case of the *Hypericum perforatum* oil extract, the HPLC-UV chromatogram revealed characteristic peaks corresponding to hypericin-related compounds, including pseudohypericin and hypericin, detected at 590 nm.

### 4.4. Development of Alginate-Based Emulgel

#### 4.4.1. Emulgel Formulation and Preparation

The alginate-based emulgel was formulated by combining a hydrophilic phase containing sodium alginate, hyaluronic acid, glycerin, and hydroglyceric plant extracts with a lipid phase composed of oil extracts, emulsifier, and co-emulsifier. Antioxidant, chelating, and preservative components were subsequently incorporated to ensure formulation stability and functional performance, as presented in Table 3.

Briefly, sodium alginate was dispersed in purified water under continuous stirring until complete hydration. Glycerin, hyaluronic acid, and the hydroglyceric extracts of *Calendula officinalis*, *Matricaria chamomilla*, and *Plantago major* were then incorporated into the aqueous phase. Separately, the lipid phase, consisting of *Hippophae rhamnoides* and *Hypericum perforatum* oil extracts, Olivem^®^ 1000, and Polysorbate 80, was heated to 60–70 °C and added to the aqueous phase under mechanical stirring and high-shear homogenization. After cooling below 40 °C, tocopheryl acetate, EDTA, and the preservative were added, and the formulation was mixed until a homogeneous emulgel was obtained.

#### 4.4.2. Stability and Physicochemical Characterization of Emulgel

##### Organoleptic Properties

The organoleptic characteristics of the emulgel, including appearance, color, odor, texture, consistency, and homogeneity, were evaluated by visual inspection at room temperature under normal daylight conditions. The formulation was examined for phase separation, heterogeneity, aggregates, and visible particles, while the observed characteristics were qualitatively recorded. Texture and spreadability were additionally assessed by gentle manual spreading, providing information regarding the overall quality and sensory acceptability of the formulation [72].

##### pH Measurement

The pH of the alginate-based emulgel was determined to evaluate its suitability for topical application and use as a printable matrix for 3D-printed patches [72,73]. Approximately 1 g of emulgel was dispersed in 10 mL of distilled water or phosphate-buffered saline (PBS, pH 7.4) and gently mixed to obtain a homogeneous dispersion. The pH was measured at room temperature (25 ± 2 °C) using a calibrated digital pH meter (Consort, Turnhout, Belgium). The electrode was immersed in the sample, and the measurement was recorded after stabilization. All determinations were performed in triplicate, and the results were expressed as mean ± standard deviation (SD). The obtained values were compared with the physiological skin pH range (approximately 4.5–6.5).

##### Rheological Behaviour

The rheological behavior of the emulgel formulation was evaluated using a rotational viscometer (Fungilab, Barcelona, Spain). Measurements were performed at room temperature (25 ± 2 °C) using spindle no. 7 at different rotational speeds (100, 150, and 200 rpm) to assess the flow behavior of the formulation [72,73]. All measurements were performed in triplicate, and the results were expressed as mean ± standard deviation.

##### Yield Stress Analysis

The rheological behavior of the emulgel was also evaluated using a Brookfield DV2T rotational viscometer (AMETEK Brookfield, Middleboro, MA, USA). Measurements were performed under controlled conditions using a multi-step shear protocol covering shear rates between 0.20 and 0.75 s^−1^. Each shear rate was maintained for 60 s prior to data acquisition in order to allow structural equilibration. Shear stress and viscosity data were recorded automatically by the instrument. Yield stress was estimated using the Casson rheological model according to the formula

(1) $$\sqrt{\tau }=\sqrt{\tau_{0}}+\sqrt{\eta_{c}}\sqrt{\dot{\gamma }},$$

where τ is the shear stress, $\tau_{0}$ is the Casson yield stress, $\eta_{c}$ is the Casson plastic viscosity and $\dot{\gamma }$ is the shear rate. The linearized form of the Casson equation was fitted by linear regression, and the Casson yield stress (*τ*_0_) and plastic viscosity ($\eta_{c}$) were calculated from the intercept and slope of the resulting regression line, respectively [31,32,33].

##### Structural Recovery After Extrusion

Structural recovery is considered a critical rheological parameter for extrusion-based systems because it reflects the ability of a material to rebuild its internal structure after experiencing high shear stresses during extrusion. Rapid viscosity recovery contributes to shape fidelity and structural stability after deposition [74,75,76,77].

A simplified three-step thixotropic recovery test was performed using a rotational viscometer (Smart, Fungilab S.A., Barcelona, Spain). The emulgel was initially maintained at 10 rpm for 60 s to establish its initial structural state. Subsequently, the sample was subjected to high shear conditions (100 rpm for 300 s) to simulate the shear-induced structural disruption occurring during extrusion. Following cessation of the high-shear step structural recovery was monitored at 10 rpm for up to 600 s, with viscosity measurements recorded at 0, 60, 180, 300 and 600 s (Table S7 from Supplementary Materials) and calculated according to the following equation:

(2) $$Recovery\;(\%)=\frac{\eta_{t}}{\eta_{i}}\times 100,$$

where $\eta_{i}$ is the initial viscosity, and $\eta_{t}$ is the viscosity measured at time t during recovery.

The structural retention under high shear was calculated according to the following equation:

(3) $$Structural\;Retention\;(\%)=\frac{\eta_{HS}}{\eta_{i}}\times 100,$$

where $\eta_{HS}$ is the viscosity measured under high shear conditions, and $\eta_{i}$ is the initial viscosity.

The percentage of viscosity loss induced by high-shear treatment was determined using the following equation:

(4) $$Viscosity\;loss\;(\%)=\frac{\eta_{i}-\eta_{high\;shear}}{\eta_{i}}\times 100,$$

##### Spreadability

The spreadability of the emulgel was evaluated using the Ojeda–Arbusa method [35]. Briefly, 1 g of emulgel was placed between two glass plates, and different weights (10, 20, 30, 50, 100, 150, and 200 g) were sequentially applied to the upper plate for 1 min to allow uniform spreading. After removal of the applied weight, the diameter of the spread emulgel layer was measured using a digital micrometer (Mitutoyo, Kawasaki, Japan). Spreadability was calculated using the following equation:

(5) $$Si=\frac{\pi {d_{i}}^{2}}{4},$$

where $S_{i}$ (mm^2^) represents the spreading area obtained when the mass i (g) is applied, and $d_{i}$ is the diameter (mm) of the sample.

##### Physical Stability Studies

The physical stability of the emulgel was evaluated using accelerated and stress stability tests, including centrifugation, thermal stress, freeze–thaw cycles, and creaming index determination.

Centrifugation Test

An accelerated stability test was performed by centrifuging the emulgel at 3000 rpm for 30 min at room temperature using a benchtop centrifuge (Hettich EBA 200, Hettich, Germany). Two samples (5 g each) were visually examined for phase separation, creaming, sedimentation, or coalescence after centrifugation.

Thermal Stress Test

Thermal stability was assessed by storing the emulgel samples at 4 ± 2 °C, 20 ± 2 °C, and 37 ± 2 °C for 14 days. The samples were periodically evaluated for changes in appearance, color, odor, consistency, homogeneity, and phase separation.

Freeze–Thaw Cycles

The resistance of the formulation to temperature fluctuations was evaluated using freeze–thaw cycles [78]. Two samples (5 g each) were subjected to six consecutive cycles consisting of storage at −40 °C for 24 h followed by thawing at +40 °C for 24 h. After each cycle, the samples were visually examined for phase separation and changes in texture, consistency, or homogeneity.

Creaming Index

The creaming index was determined to further evaluate the physical stability of the emulgel. A 10 g sample was transferred into graduated glass tubes and stored undisturbed at room temperature. At predetermined time intervals (0, 7, 14, 21, and 30 days), the height of the separated phase ($H_{s}$) and the total sample height ($H_{t}$) were measured. The creaming index was calculated according to the following equation:

(6) $$CI(\%)=\frac{H_{s}}{H_{t}}\times 100,$$

where $H_{s}$ represents the height of the separated layer, and $H_{t}$ represents the total height of the emulgel sample.

### 4.5. Fabrication and Pharmacotechnical Characterisation of 3D-Printed Patches

#### 4.5.1. Three-Dimensional Printing of Patches

**Figure 10:**
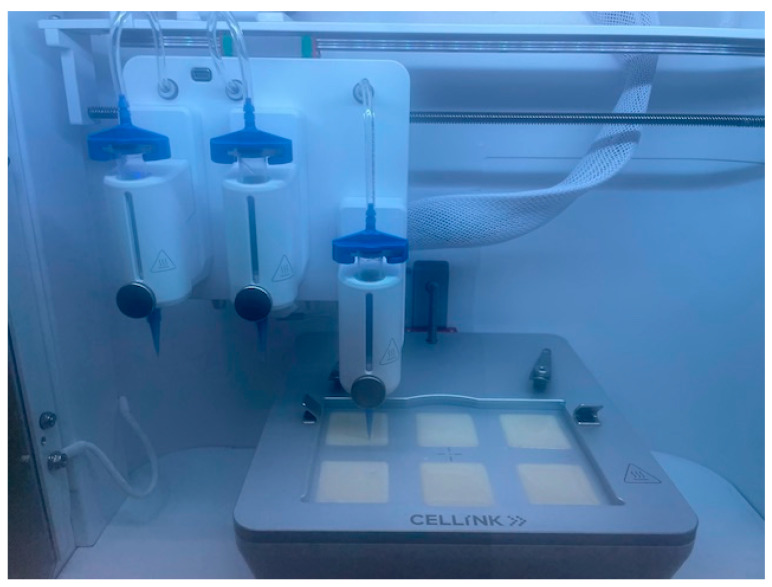
Three-dimensional printing of alginate-based patches during the fabrication process.

The patches were fabricated using a 3D Cellink BIO X bioprinter (CELLINK, Gothenburg, Sweden), equipped with a pneumatic extrusion system. The printing process was performed at room temperature using a 3 mL syringe fitted with a blue nozzle. The printing parameters were set as follows: an extrusion pressure of 13 kPa, a printing speed of 13.1 mm/s, and a layer height of 0.2 mm. A grid infill pattern with square geometry and 100% infill density was employed to ensure structural integrity. The designed patch dimensions were 30 × 30 × 1 mm. The patches were printed in batches of six under identical conditions (Figure 10).

#### 4.5.2. Crosslinking Procedure

Crosslinking of the 3D-printed patches was performed using a 2% (*w*/*v*) calcium chloride solution at room temperature. Initially, the patches were sprayed with the crosslinking solution to ensure uniform surface gelation, followed by immersion in the same solution for 5 min to complete the crosslinking process. Subsequently, the patches were rinsed with distilled water to remove excess calcium ions and stored in airtight containers at refrigerated conditions, with a few drops of distilled water added to maintain hydration until further use [79,80].

#### 4.5.3. Weight Uniformity

For the assessment of weight uniformity, 20 films from each batch of patches were individually weighed using a calibrated analytical balance. All measurements were performed in triplicate, and the values were recorded to calculate the mean weight, along with standard deviation (SD), in order to evaluate the consistency and reproducibility of the fabricated patches [81].

#### 4.5.4. Dimensional Characterisation

For dimensional characterization, twenty films from each batch were assessed for thickness, length, and width, using a digital Mitutoyo micrometer (Mitutoyo, Kawasaki, Japan). Measurements were performed at multiple points on each film to account for potential variability, and all determinations were carried out in triplicate. Mean values with standard deviations (SDs) were calculated for each batch to evaluate uniformity, reproducibility, and overall geometric consistency of the printed patches [82].

#### 4.5.5. Folding Endurance

Folding endurance was determined to evaluate the flexibility of the patches [83]. The 3D-bioprinted patches were repeatedly folded at the same location until visible cracking or breakage occurred, or up to a maximum of 300 folds, which was considered the upper limit of the method. The number of folds required to cause rupture was recorded as the folding endurance value. All measurements were performed in triplicate and expressed as mean ± standard deviation (SD), when applicable.

#### 4.5.6. Loss on Drying

The moisture content of the alginate-based hydrocolloid dressings was determined using a gravimetric method [81,84]. Dressing samples were cut into small pieces and accurately weighed to obtain the initial mass (W_1_). The specimens were then dried in a laboratory oven at 105 °C until constant weight was achieved (typically 24 h). After cooling in a desiccator to room temperature, the dried mass (W_2_) was recorded. The moisture content was calculated using the following equation:

(7) $$LOD(\%)=(W_{1}-W_{2})/W_{1}\times 100,$$

where W_1_ represents the initial mass of the sample and W_2_ the mass after drying.

All measurements were performed in triplicate, and the results were expressed as mean values ± standard deviation.

#### 4.5.7. Surface pH

The surface pH of the prepared patches was determined to evaluate their skin compatibility. Measurements were performed under two hydrated conditions, namely in distilled water and in phosphate-buffered solution (PBS, pH 7.4), to assess the behavior of the patches in aqueous and physiologically relevant environments.

For measurements in distilled water, patch samples were placed in Petri dishes and moistened with a few drops of distilled water to allow surface hydration and equilibration. For measurements in PBS, the samples were immersed in 5 mL of phosphate-buffered solution (pH 7.4) and allowed to swell, according to previously reported methods [40]. The pH was measured at predetermined time intervals (2, 4, and 6 h) by placing the electrode of a calibrated digital pH meter (WTW 7110, Xylem Analytics, Weilheim, Germany) directly onto the hydrated patch surface.

All determinations were performed in triplicate, and the results were expressed as mean ± standard deviation (SD).

#### 4.5.8. Swelling Capacity

The swelling capacity of the alginate-based 3D-printed patches was evaluated according to EN 13726-1 [85]. Square samples (5 × 5 cm) were accurately weighed to determine the initial dry mass (m_1_), then fully immersed in 0.9% NaCl solution at 37 °C for 30 min to simulate wound exudate conditions. After removal, the samples were allowed to drain freely for 30 s, and the swollen mass (m_2_) was recorded.

The swelling capacity (SC) was calculated with the formula

(8) $$SC=\frac{m_{2}-m_{1}}{A},$$

where A represents the sample surface area, m_1_ represents the initial dry mass, and m_2_ represents the swollen mass. The results were expressed as g of absorbed fluid per 100 cm^2^ of dressing.

All measurements were performed in triplicate, and results were reported as mean ± standard deviation (SD).

#### 4.5.9. Water Vapor Transmission Rate (WVTR)

The water vapor transmission rate (WVTR) of the 3D-printed patches was determined using a gravimetric method. Briefly, each patch was fixed over the opening of a glass vial containing distilled water, ensuring complete coverage of the exposed area. The edges were carefully sealed to prevent vapor loss through the sides. The initial weight of each assembly was recorded, and the vials were maintained under controlled conditions for 24 h. After this period, the final weight was recorded, and the mass loss was attributed to water vapor transmission through the patch.

WVTR was calculated according to the following equation:

(9) $$WVTR=\frac{\Delta W}{A\times t},$$

where ΔW represents the weight loss of the vial assembly (g), A  is the exposed surface area of the patch (m^2^), and t’  is the exposure time (24 h). The results were expressed as g/m^2^/24 h [82]. All measurements were performed in triplicate, and the results were reported as mean ± standard deviation.

### 4.6. Phytochemical Analysis

#### Total Polyphenol Content

The total polyphenol contents (TPCs) of the alginate-based emulgel and the corresponding 3D-printed patches were determined using the Folin–Ciocalteu colorimetric method, with slight modifications [86,87]. Briefly, 2 g of sample (emulgel or patch) were extracted with 25 mL of 80% ethanol under mechanical stirring for 10 min, followed by sonication at 40 °C for 30 min and centrifugation at 5000 rpm for 10 min. In the case of the patches, the samples were previously cut into small pieces to facilitate extraction. The resulting supernatant was filtered and adjusted to a final volume of 25 mL.

An aliquot of the obtained extract (0.5 mL) was mixed with 2.5 mL of 10% Folin–Ciocalteu reagent, and after 5 min, 2.0 mL of 7.5% sodium carbonate solution was added. The mixture was incubated in the dark for 30 min at room temperature, and the absorbance was measured at 765 nm using a VWR UV-6300 PC spectrophotometer (VWR International, Vienna, Austria). Gallic acid was used as the calibration standard, and the results were expressed as milligrams of gallic acid equivalents per gram of sample (mg GAE/g). All measurements were performed in triplicate.

### 4.7. Structural Characterization

#### 4.7.1. Scanning Electron Microscopy (SEM)

The morphology of the alginate-based emulgel and Ca^2+^-crosslinked alginate patch was analyzed by a Scanning electron microscopy (SEM) technique using a Nova Nano SEM 630 instrument (FEI Company, Hillsboro, OR, USA). The alginate-based emulgel sample was coated with a thin layer of gold (Au) prior to imaging in order to improve surface conductivity and image quality. The SEM analysis of the alginate patch was performed under high vacuum conditions at an accelerating voltage of 10 kV, utilizing a low probe current to prevent beam damage and sample charging. For SEM analysis, the samples were first lyophilized and subsequently coated with a thin layer of gold (Au) to improve surface conductivity and imaging quality.

#### 4.7.2. Scanning Electron Microscopy Coupled with Energy-Dispersive X-Ray Spectroscopy (SEM–EDX)

The distribution of calcium ions within the crosslinked alginate matrix was investigated using a Smart Insight AMETEK Energy-Dispersive X-ray Spectroscopy (EDS) system coupled with SEM imaging. The analysis was performed to confirm the incorporation and homogeneous distribution of calcium following the ionic crosslinking process.

#### 4.7.3. Mechanical Characterization by AFM Nanoindentation

Young’s modulus of the Ca^2+^-crosslinked alginate patch was determined by AFM-based nanoindentation using an Ntegra Atomic Force Microscope (NT-MDT Spectrum Instruments). Indentation experiments were performed using soft silicon–pyrex nitride probes (PNP) with a nominal spring constant of 0.06 N/m and pyramidal tips with a half-angle of 35°. Prior to measurements, the inverse optical lever sensitivity was calibrated using a sapphire reference surface. Force–distance curves were acquired at multiple locations on the patch surface. The indentation data were fitted using the DMT spherical contact mechanics model, and Young’s modulus values were calculated using the Force Curve Processor software V3.5. The reported modulus corresponds to the average of 20 independent measurements. It should be noted that the AFM nanoindentation measurements were performed in air; therefore, the obtained Young modulus values represent the mechanical response of the partially dehydrated surface layer of the hydrogel rather than the fully hydrated bulk material [56].

#### 4.7.4. Dynamic Light Scattering (D.L.S.) Analysis

The hydrodynamic diameter and surface charge of the colloidal dispersions were characterized using a Delsa Nano C instrument from Beckman Coulter, Brea, CA, USA, by dynamic light scattering (DLS) and electrophoretic light scattering (ELS). Particles were illuminated by a dual 30 mW laser diode of 658 nm, producing time-dependent fluctuations in the intensity of the laser light. The scattered light was collected at 165° for size measurements and 15° for zeta potential measurements (diluted concentration samples) and then measured by a highly sensitive detector. Prior to analysis, 0.1 g of emulgel was dispersed in 10 mL of distilled water (10 mg/mL) and gently homogenized to obtain a measurable dispersion. Both measurement types (DLS and ELS) were performed at room temperature, each sample measurement was performed in triplicate, and for data analysis, the Delsa^TM^Nano 3.73 software was used.

#### 4.7.5. Fourier Transform Infrared Spectroscopy (FTIR)

Fourier-transform infrared spectroscopy (FTIR) was performed to investigate the chemical interactions and structural modifications occurring during the transformation of the alginate-based emulgel into Ca^2+^-crosslinked 3D-printed patches. ATR-FTIR spectra of the emulgel and the corresponding crosslinked patches were recorded using a Tensor 27 spectrometer (Bruker Optics, Karlsruhe, Germany) equipped with a Platinum ATR accessory containing a single-reflection diamond crystal. Spectra were collected after 64 scans, at a spectral resolution of 4 cm^−1^, within the 4000–370 cm^−1^ range, and processed using OPUS 6.0 software.

### 4.8. Functional and Biological Evaluation

#### 4.8.1. Antioxidant Activity

The antioxidant activity of the alginate-based emulgel and the corresponding 3D-printed patches were evaluated using the 2, 2′-azinobis (3-ethylbenzothiazoline-6-sulfonic acid) (ABTS) radical cation decolorization assay, with slight modifications [88]. Briefly, 2 g of sample (emulgel or patches) were extracted with 25 mL of 80% ethanol under mechanical stirring for 10 min, followed by sonication at 40 °C for 30 min and centrifugation at 5000 rpm for 10 min. Patch samples were previously cut into small pieces to facilitate extraction. The obtained supernatants were filtered and used for analysis.

The ABTS•+ radical cation was generated by mixing equal volumes of ABTS solution (7 mM) and potassium persulfate solution (2.45 mM), followed by incubation in the dark at room temperature for 12–16 h. Prior to analysis, the ABTS•+ solution was diluted with ethanol to an absorbance of 0.70 ± 0.02 at 734 nm. For the assay, 50 µL of sample extract was mixed with 4.95 mL of ABTS•+ solution, and the absorbance decrease was measured at 734 nm after 6 min, using a VWR UV-6300 PC spectrophotometer (VWR International, Vienna, Austria).

Radical scavenging activity was calculated according to the following equation:

(10) $$\%\;inhibition=\frac{A_{0}-A_{s}}{A_{0}}\times 100,$$

where $A_{0}$ represents the absorbance of the control, and $A_{s}$ represents the absorbance of the sample. The control solution was prepared under the same conditions by replacing the sample extract with an equal volume of ethanol. Antioxidant activity was expressed as IC_50_ values, calculated based on the final concentrations in the reaction mixture, and as Trolox-equivalent concentrations. All measurements were performed in triplicate, and the results were expressed as mean ± standard deviation (SD).

#### 4.8.2. In Vitro Polyphenol Release

The in vitro release of polyphenols from the 3D-printed patches was evaluated using a USP Apparatus 2 (paddle method) [89,90]. Each patch was fixed at the bottom of the dissolution vessel using an inert stainless-steel sinker to prevent floating and maintain a constant exposed surface area. Although USP <725> recommends Apparatus 5 for transdermal systems, Apparatus 2 was used as an adapted exploratory setup compatible with the available laboratory equipment [89].

The dissolution medium consisted of 500 mL PBS (pH 7.4)–ethanol (70:30, *v*/*v*), maintained at 37.0 ± 0.5 °C, with paddle rotation set at 75 rpm. The hydroalcoholic medium was selected to ensure adequate solubility of both hydrophilic and lipophilic phenolic constituents. At predetermined intervals (2, 4, 5, 6, 20, and 24 h), 3 mL samples were withdrawn without medium replacement, and cumulative release values were corrected for sampling-induced volume loss. The collected samples were filtered through 0.45 µm membrane filters, and released polyphenols were quantified using the Folin–Ciocalteu method at 765 nm, based on a gallic acid calibration curve.

The results were expressed as released polyphenol amount (µg GAE) and cumulative percentage release, calculated relative to the estimated total polyphenol content of each patch based on the TPC values of the individual extracts and the formulation composition (Table S4). All experiments were performed in triplicate, and data were reported as mean ± standard deviation (SD).

Kinetic analysis was performed by fitting the cumulative release data to zero-order, first-order, Higuchi, Korsmeyer–Peppas, and Weibull models using the linear regression of the appropriately linearized equations. For the Korsmeyer–Peppas model, only data points with *M*t/*M*∞ < 0.60 were included. Goodness of fit was evaluated using R^2^, and the release mechanism was inferred from the Korsmeyer–Peppas exponent (*n*) and the Weibull shape parameter (*b*).

#### 4.8.3. Biocompatibility Assessment

The biocompatibility of the 3D-printed patches was evaluated using HaCaT cells, an immortalized human epidermal keratinocyte cell line obtained from Cell Line Service GmbH (catalog no. 330493, Eppelheim, Germany). Cells were cultured in Dulbecco’s Modified Eagle’s Medium (DMEM) supplemented with 10% fetal bovine serum, 2 mM L-glutamine, 100 U/mL penicillin, and 100 μg/mL streptomycin, and maintained at 37 °C in a humidified atmosphere containing 5% CO_2_.

Cell viability was assessed using the CellTiter 96^®^ AQueous One Solution Cell Proliferation Assay (Promega, Madison, WI, USA), an MTS-based colorimetric method. Briefly, HaCaT cells were seeded in 96-well plates at a density of 1 × 10^4^ cells/well in 100 µL of culture medium and incubated for 24 h. The culture medium was then removed, and the cells were exposed to increasing concentrations of the patch extract for 24, 48, and 72 h. Untreated cells were used as controls.

After treatment, 20 µL of MTS/PES reagent was added to each well, and the plates were incubated for 4 h at 37 °C. The absorbance of the resulting formazan product was measured at 492 nm using a DYNEX Technologies microplate reader (DYNEX Technologies MRS, Chantilly, VA, USA). Cell viability was calculated relative to untreated control cells, considered 100% viable, according to the following equation:

(11) $$\mathrm{Cell}\;\mathrm{viability}(\%)=\frac{A_{\mathrm{treated}\;\mathrm{cells}}-A_{\mathrm{culture}\;\mathrm{medium}}}{A_{\mathrm{untreated}\;\mathrm{cells}}-A_{\mathrm{culture}\;\mathrm{medium}}}\times 100,$$

All experiments were performed in triplicate, and the results were expressed as mean ± standard deviation (SD) [91,92].

## Figures and Tables

**Table 1:** The surface pH of the 3D-printed patches measured in distilled water at different time intervals (2, 4 and 6 h). Data are expressed as mean ± SD (n = 3).

Sample	Time (h)		
	2	4	6
Patches 1 (lot 1)	6.28	6.31	6.29
	6.36	6.39	6.33
	6.32	6.35	6.31
Mean ± SD	6.32 ± 0.04	6.35 ± 0.04	6.31 ± 0.02
Patches 2 (lot 2)	6.37	6.39	6.44
	6.41	6.35	6.43
	6.39	6.37	6.43
Mean ± SD	6.39 ± 0.02	6.37 ± 0.02	6.41 ± 0.02

**Table 2:** The surface pH of the 3D-printed patches measured in phosphate-buffered solution (pH 7.4) at different time intervals (2, 4 and 6 h). Data are expressed as mean ± SD (n = 3).

Sample	Time (h)		
	2	4	6
Patches 1 (lot 1)	7.37	7.33	7.33
	7.35	7.37	7.31
	7.33	7.35	7.35
Mean ± SD	7.35 ± 0.02	7.35 ± 0.02	7.33 ± 0.02
Patches 2 (lot 2)	7.29	7.35	7.33
	7.33	7.34	7.35
	7.31	7.33	7.33
Mean ± SD	7.31 ± 0.02	7.34 ± 0.01	7.33 ± 0.02

**Table 3:** Composition of the alginate-based emulgel formulation.

Phase	Ingredient	Amount (% *w*/*w*)	Function
Phase A	Sodium alginate	3.00	Gelling agent
	Hyaluronic acid	0.20	Humectant; tissue regeneration support
	Glycerin	3.00	Humectant
	*Calendula officinalis* hydroglyceric extract	3.00	Plant-derived bioactive extract
	*Matricaria chamomilla* hydroglyceric extract	3.00	Plant-derived bioactive extract
	*Plantago major* hydroglyceric extract	3.00	Plant-derived bioactive extract
Phase B	*Hippophae rhamnoides* oil extract	2.00	Lipophilic bioactive extract
	*Hypericum perforatum* oil extract	2.00	Lipophilic bioactive extract
	Cetearyl Olivate (and) Sorbitan Olivate *	0.20	Emulsifier
	Tween 80 **	1.00	Co-emulsifier
Phase C	Tocopheryl acetate ***	0.20	Antioxidant
	Benzyl Alcohol (and) Dehydroacetic Acid ****	0.80	Preservative
	EDTA	0.10	Chelating agent
Phase D	Purified water	Up to 100 g	Vehicle

* Ollivem^®^1000; ** Polysorbate 80; *** Vitamin E; **** Cosgard.

## Data Availability

The data supporting the findings of this study are available within the article and its Supplementary Materials.

## Supplementary Materials

The following supporting information can be downloaded at https://www.mdpi.com/article/10.3390/gels12060541/s1. Figure S1: *Calendula Officinalis* HPLC chromatogram; Figure S2: *Matricaria chamomilla* HPLC chromatogram; Figure S3: *Plantago major* HPLC chromatogram; Figure S4: *Hippophae rhamnoides* HPLC chromatogram; Figure S5: *Hypericum perforatum* HPLC chromatogram; Figure S6: Variation of pH values of the emulgel over 30 days of storage; Figure S7: Linearized Casson model obtained from rheological measurements of the alginate-based emulgel; Figure S8: Representative AFM force–distance curves acquired on the surface of the Ca^2+^-crosslinked alginate patch during approach and retraction cycles. The observed hysteresis between the two curves reflects adhesive interactions and the viscoelastic nature of the hydrogel surface. These force curves were used for Young’s modulus estimation through DMT contact mechanics fitting; Figure S9A: Instrument-recorded DLS intensity-based size distribution of the alginate-based emulgel formulation, showing the apparent hydrodynamic diameter profile of the dispersed domains present within the system; Figure S9B: Instrument-recorded zeta potential distribution of the alginate-based emulgel formulation; Table S1: Experimental shear rate and shear stress values together with the corresponding linearized Casson parameters used for determination of the Casson yield stress and plastic viscosity of the alginate-based emulgel; Table S2: Rheological indicators derived from the three-step recovery test; Table S3: Effect of storage temperature on the physical stability of the emulgel; Table S4: Freeze-thaw stability of the emulgel; Table S5: Possible assignment of the spectral bands for the emulgel sample and the sodium alginate patch sample; Table S6: Kinetic modeling of polyphenol release from 3D-printed alginate emulgel patches; Table S7: Structural recovery of the emulgel following high-shear treatment.
