# Supplementary material for: Multifunctional 3D-Printed Alginate Emulgel Patches Incorporating Plant Extracts for Potential Burn Wound Applications
Source: Gels. 2026 Jun 17;12(6):541. doi: 10.3390/gels12060541 (PMC13299215; doi:10.3390/gels12060541)
Supplement: Supplementary file 1 [file gels-12-00541-s001.zip › gels-4365340-supplementary.pdf]

SUPPLEMENTARY MATERIALS

Manuscript ID: gels-4365340

Multifunctional 3D-Printed Alginate Emulgel Patches Incorporating Plant Extracts for Potential Burn Wound Applications

Roxana Colette Sandulovici[1]†, Ion Mircioiu[1]†, Mariana Panțuroiu[1], *Corneliu Dan Blendea*[2], Mirela Claudia Rîmbu[1], Daniel Cord[1], Carmen Elisabetta Manea[1,3], Carmen Marinela Mihăilescu[1,4,5], Mirela Antonela Mihăilă[1,6], Iulian Sârbu[1], Horia Sebastian Iliescu[1], Manuel Ovidiu Amzoiu[7], Adina Boldeiu[4], Vasilica Țucureanu[4], Oana Brîncoveanu[4], Luiza Mădălina Cima[1] and Mona Luciana Gălățanu[1]

[1] Faculty of Pharmacy, Titu Maiorescu University, Bucharest, Romania

[2] Faculty of Medicine, Titu Maiorescu University, Bucharest, Romania

[3] Horia Hulubei National Institute for R&D in Physics and Nuclear Engineering (IFIN-HH), Măgurele, Romania

[4] National Institute for Research and Development in Microtechnologies (IMT), Bucharest, Romania

[5] Doctoral School, University of Medicine and Pharmacy of Craiova, Craiova, Romania

[6] Stefan S. Nicolau Institute of Virology, Bucharest, Romania

[7] Faculty of Pharmacy, University of Medicine and Pharmacy of Craiova, Craiova, Romania

Correspondence: mariana.panturoiu@prof.utm.ro danblendea@gmail.com

† These authors contributed equally to this work.

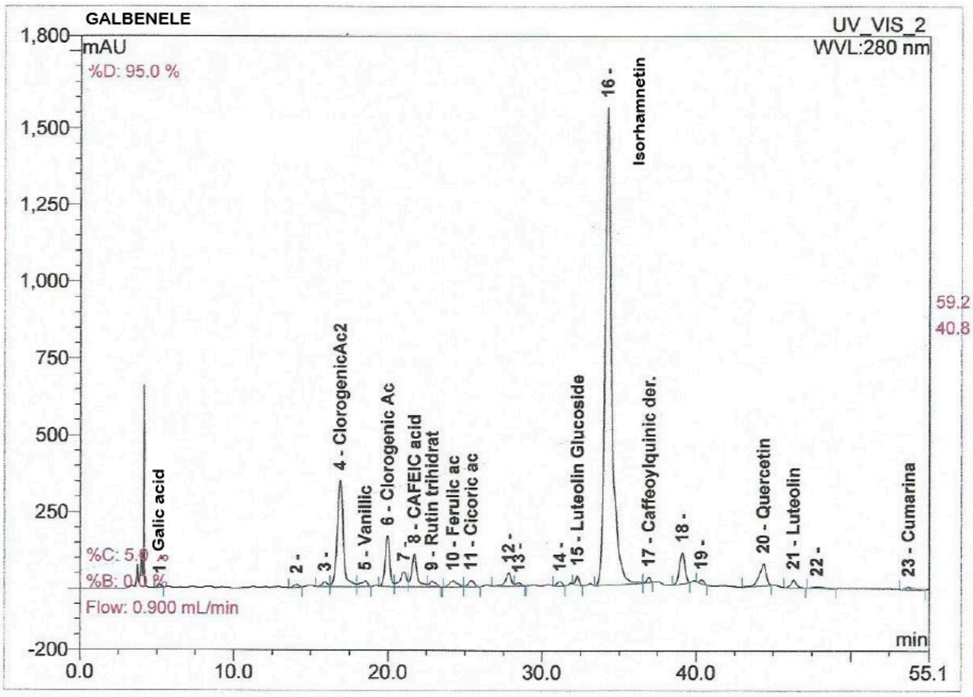

Figure S1. *Calendula Officinalis* HPLC chromatogram.

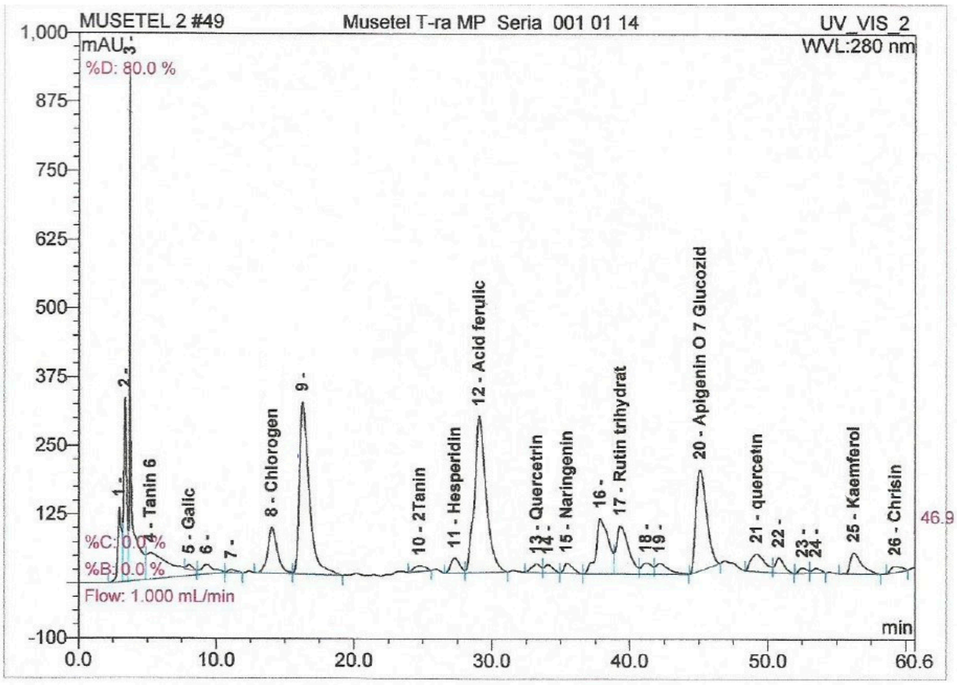

Figure S2. *Matricaria chamomilla* HPLC chromatogram.

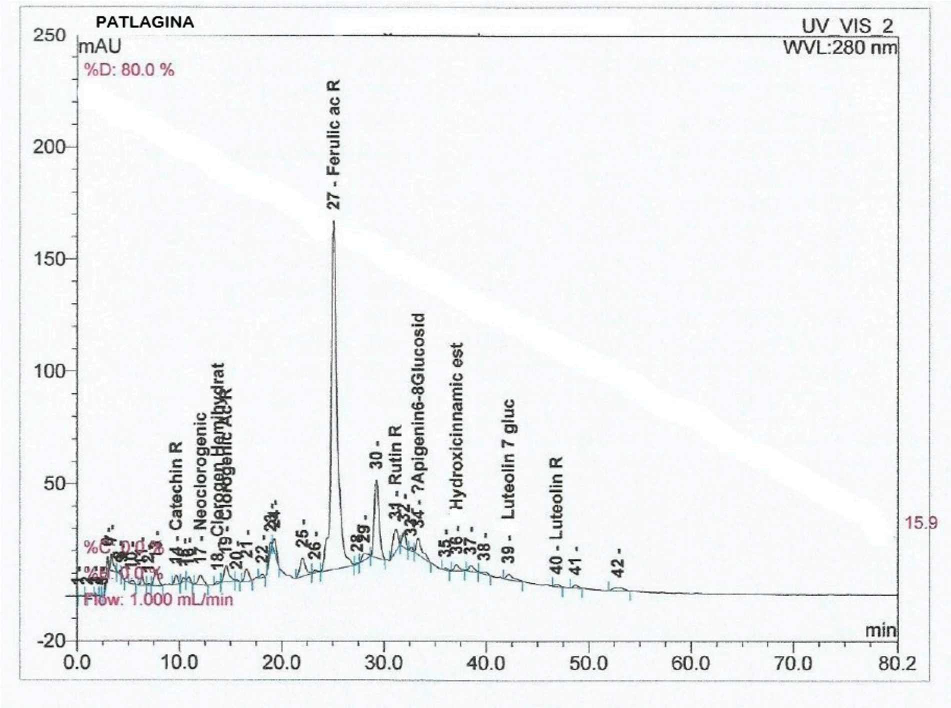

Figure S3. *Plantago major* HPLC chromatogram.

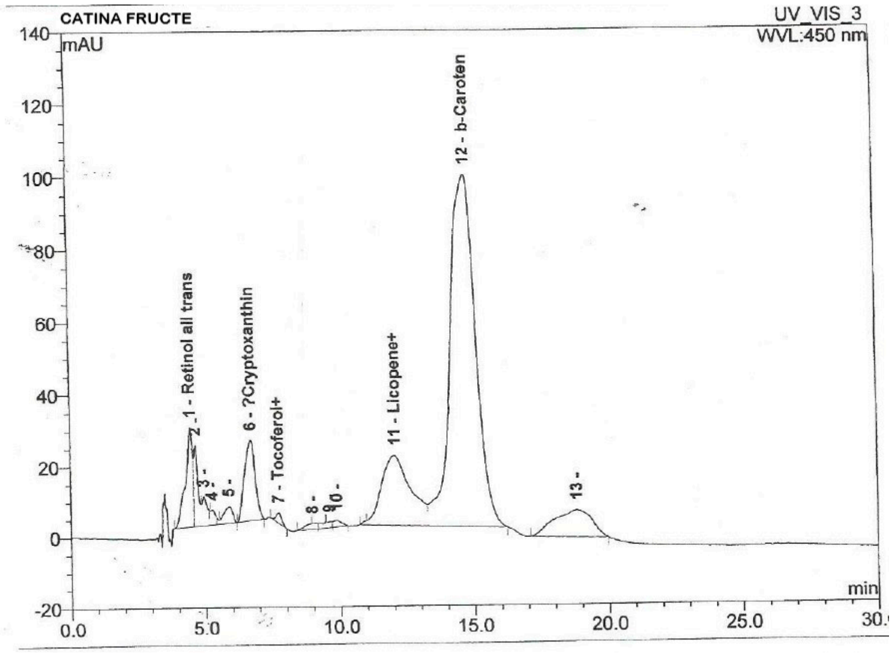

Figure S4. *Hippophae rhamnoides* HPLC chromatogram.

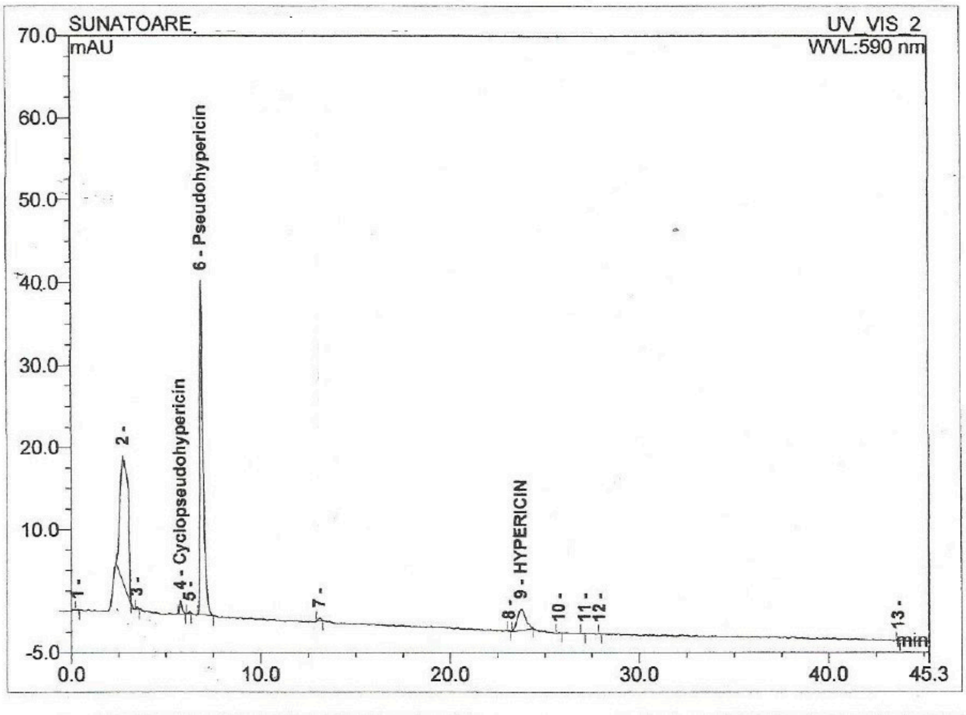

Figure S5. *Hypericum perforatum* HPLC chromatogram.

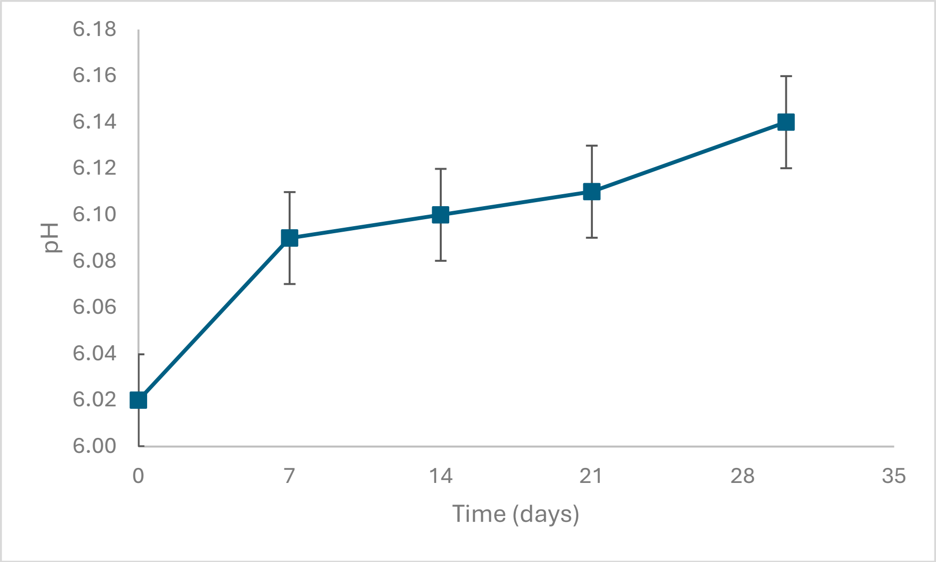

Figure S6. Variation of pH values of the emulgel over 30 days of storage. Data are expressed as mean ± SD (n = 3).

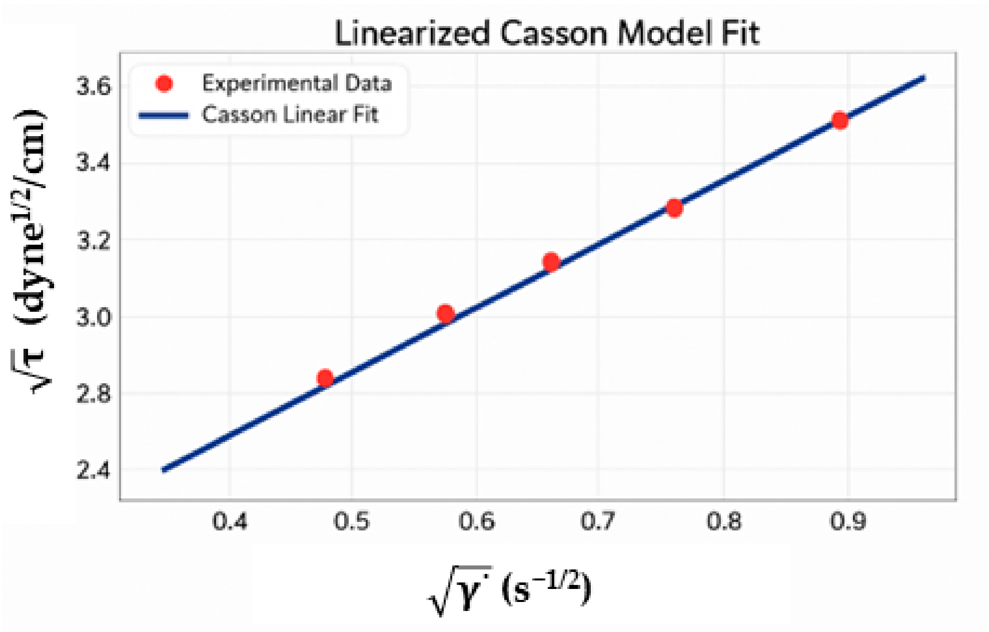

Figure S7. Linearized Casson model obtained from rheological measurements of the alginate-based emulgel. The intercept of the regression line was used to calculate the Casson yield stress (τ_0), while the slope was used to determine the Casson plastic viscosity (ηc).

Table S1. Experimental shear rate and shear stress values together with the corresponding linearized Casson parameters used for determination of the Casson yield stress and plastic viscosity of the alginate-based emulgel.

**Shear Rate, $\dot{\gamma}$ (s^{-1})**	**$\sqrt{\dot{\gamma}}$ ($s^{-1/2}$)**	**Shear Stress, τ (dyne/cm^2)**	**$\sqrt{\tau}$ (dyne$^{1/2}$/cm)**
0.75	0.8660	11.91	3.4511
0.55	0.7416	10.35	3.2171
0.42	0.6481	9.80	3.1305
0.32	0.5657	8.20	2.8636
0.20	0.4472	7.00	2.6458

Table S2. Rheological indicators derived from the three-step recovery test

Parameter	Value (%)
Structural retention under high shear	58.8
Viscosity loss during high shear	41.2
Structural recovery after 600 s	85.1

Table S3. Effect of storage temperature on the physical stability of the emulgel

Parameter	4 ± 2° C	25 ± 2° C	37 ± 2° C
Appearance	No visible changes	No visible changes	Slight decrease in consistency
Color	Unchanged	Unchanged	Unchanged
Odor			
Homogeneity	Characteristic, unchanged	Characteristic, unchanged	Characteristic, unchanged
Texture	Homogenous	Homogenous	Slightly reduced homogeneity
Consistency	Smooth	Smooth	Slightly less firm
Spreadability	Unchanged	Slightly increased	Increased
Stability assessment	Stable	Stable	Acceptable stability

Table S4. Freeze-thaw stability of the emulgel

Cycle number	Condition (°C)	Observations
1	– 40 → + 40	No phase separation observed
2	– 40 → + 40	No significant changes observed
3	– 40 → + 40	Slight decrease in viscosity, no separation

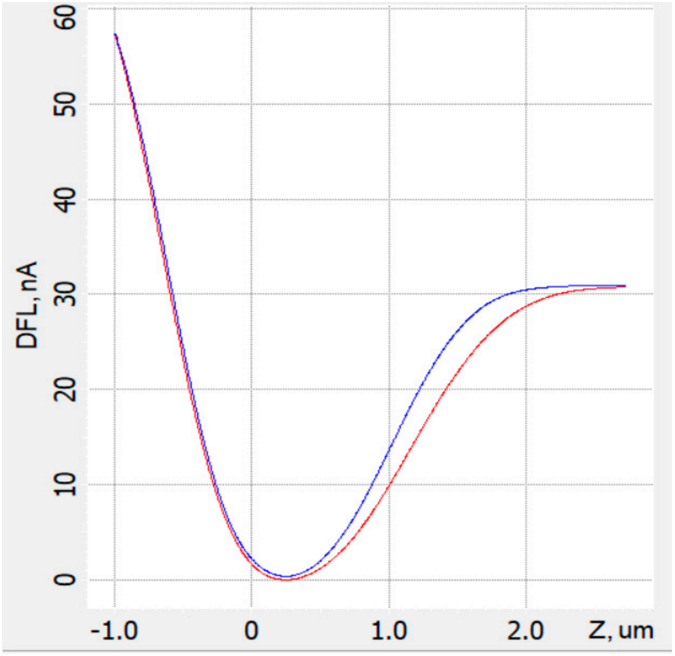

Figure S8. Representative AFM force–distance curves acquired on the surface of the Ca^{2+}-crosslinked alginate patch during approach and retraction cycles. The observed hysteresis between the two curves reflects adhesive interactions and the viscoelastic nature of the hydrogel surface. These force curves were used for Young's modulus estimation through DMT contact mechanics fitting.

Version 3.73 / 2.30

Intensity Distribution

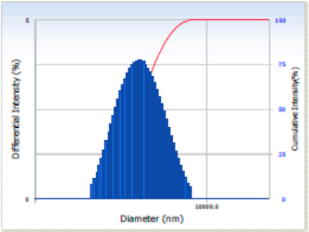

ACF

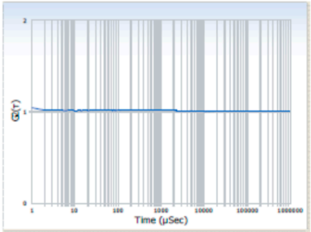

Distribution Results (Contin)

Peak	Diameter (nm)	Std. Dev.
1	4,995.0	1,206.7
2	0.0	0.0
3	0.0	0.0
4	0.0	0.0
5	0.0	0.0
Average	4,995.0	1,206.7

Residual : 1.475e-003 (O.K)

Cumulants Results

Diameter	(d)	: 8763.9	(nm)
Polydispersity Index	(P.I.)	: -11.132	
Diffusion Const.	(D)	: 5.627e-010	(cm^2/sec)
Molecular Weight		: 3.158e+012	

Measurement Condition

Temperature	: 25.1	(°C)
Diluent Name	: WATER	
Refractive Index	: 1.3328	
Viscosity	: 0.8858	(cP)
Scattering Intensity	: 28316	(cps)
Attenuator 1	: 65.58	(%)

Intensity Distribution Table

d (nm)	f(%)	f(cum.%)	d (nm)	f(%)	f(cum.%)	d (nm)	f(%)	f(cum.%)	d (nm)	f(%)	f(cum.%)
1530.0	0.0	0.0	2928.2	0.6	1.0	5604.0	3.4	72.6	10725.1	0.0	100.0
1570.2	0.0	0.0	3005.2	0.7	1.7	5751.4	3.2	75.9	11007.2	0.0	100.0
1611.5	0.0	0.0	3084.2	0.9	2.7	5902.7	3.1	78.9	11296.8	0.0	100.0
1653.9	0.0	0.0	3165.4	1.2	3.8	6058.0	2.9	81.8	11593.9	0.0	100.0
1697.4	0.0	0.0	3248.6	1.4	5.2	6217.3	2.7	84.5	11898.9	0.0	100.0
1742.1	0.0	0.0	3334.1	1.6	6.8	6380.9	2.4	86.9	12211.9	0.0	100.0
1787.9	0.0	0.0	3421.8	1.9	8.6	6548.7	2.2	89.1	12533.1	0.0	100.0
1835.0	0.0	0.0	3511.8	2.1	10.7	6721.0	2.0	91.1	12862.8	0.0	100.0
1883.2	0.0	0.0	3604.2	2.3	13.1	6897.8	1.8	92.9	13201.2	0.0	100.0
1932.8	0.0	0.0	3699.0	2.6	15.7	7079.2	1.5	94.4	13548.4	0.0	100.0
1983.6	0.0	0.0	3796.3	2.8	18.5	7265.4	1.3	95.8	13904.8	0.0	100.0
2035.8	0.0	0.0	3896.1	3.0	21.5	7456.5	1.1	96.9	14270.5	0.0	100.0
2089.3	0.0	0.0	3998.6	3.2	24.7	7652.7	0.9	97.8	14645.9	0.0	100.0
2144.3	0.0	0.0	4103.8	3.4	28.0	7854.0	0.8	98.6	15031.2	0.0	100.0
2200.7	0.0	0.0	4211.7	3.5	31.6	8060.6	0.6	99.2	15426.5	0.0	100.0
2258.6	0.0	0.0	4322.5	3.7	35.2	8272.6	0.5	99.7	15832.3	0.0	100.0
2318.0	0.0	0.0	4436.2	3.8	39.0	8490.2	0.3	100.0	16248.8	0.0	100.0
2379.0	0.0	0.0	4552.9	3.8	42.8	8713.5	0.0	100.0	16676.2	0.0	100.0
2441.5	0.0	0.0	4672.7	3.9	46.7	8942.7	0.0	100.0	17114.9	0.0	100.0
2505.8	0.0	0.0	4795.6	3.9	50.5	9177.9	0.0	100.0	17565.0	0.0	100.0
2571.7	0.0	0.0	4921.7	3.9	54.4	9419.4	0.0	100.0	18027.1	0.0	100.0
2639.3	0.0	0.0	5051.2	3.8	58.2	9667.1	0.0	100.0	18501.3	0.0	100.0
2708.7	0.0	0.0	5184.1	3.8	62.0	9921.4	0.0	100.0	18987.9	0.0	100.0
2780.0	0.0	0.0	5320.4	3.7	65.7	10182.4	0.0	100.0	19487.4	0.0	100.0

Intensity Distribution Table

d (nm)	f(%)	f(cum.%)	d (nm)	f(%)	f(cum.%)	d (nm)	f(%)	f(cum.%)	d (nm)	f(%)	f(cum.%)
2853.1	0.4	0.4	5460.4	3.5	69.2	10450.2	0.0	100.0	20000.0	0.0	100.0
D (10%) : 3,479.50 (nm)			D (50%) : 4,778.40 (nm)			D (90%) : 6,624.10 (nm)					

A.

Figure S9A. Instrument-recorded DLS intensity-based size distribution of the alginate-based emulgel formulation, showing the apparent hydrodynamic diameter profile of the dispersed domains present within the system.

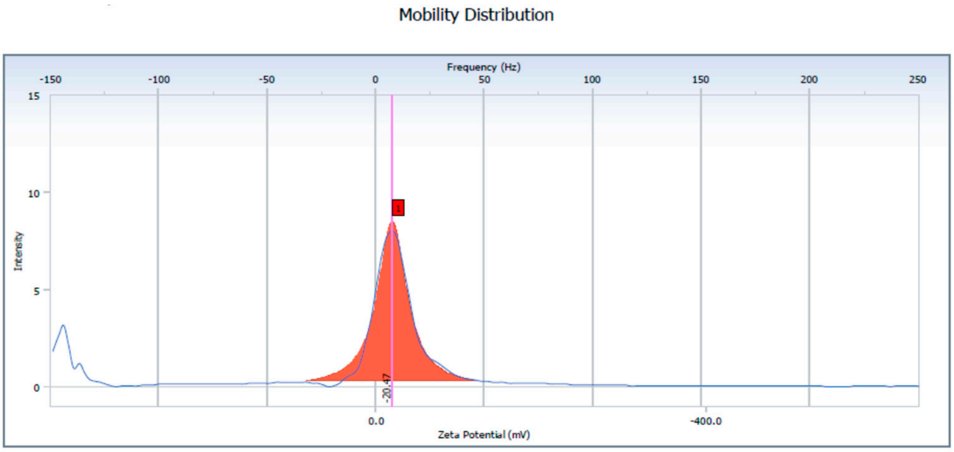

Measurement Results

Zeta Potential	: -20.47	(mV)	Doppler shift	: 7.78	(Hz)
Mobility	: -1.595e-004	(cm²/Vs)	Base Frequency	: 123.4	(Hz)
Conductivity	: 0.1504	(mS/cm)	Conversion Equation	: Smoluchowski	

Zeta Potential of Cell			Diluent Properties		
Upper Surface	: -360.12	(mV)	Diluent Name	: WATER	
Lower Surface	: 117.06	(mV)	Temperature	: 25.0	(°C)
Cell Condition			Refractive Index	: 1.3328	
Cell Type	: Flow Cell		Viscosity	: 0.8878	(cP)
Avg. Electric Field	: -10.11	(V/cm)	Dielectric Constant	: 78.2	
Avg. Current	: -0.08	(mA)			

B.

Figure S9B. Instrument-recorded zeta potential distribution of the alginate-based emulgel formulation.

Table S5. Possible assignment of the spectral bands for the emulgel sample and the sodium alginate patch sample.

Probable assignment	Characteristic for	Emulgel film Wavenumber (cm^{-1})	Patch Wavenumber (cm^{-1})
**ν(O–H)**	Sea buckthorn, alginate, glycerol, polysorbate	3299	3301
**ν(O–H)**	Sea buckthorn, alginate, glycerol, polysorbate	–	3236
**ν(=C–H)**	Sea buckthorn	3009	3009
**νas(–C–H)**	Sea buckthorn	–	2956
**νas(–C–H)**	Sea buckthorn	2923	2916
**νas(–C–H)**	Sea buckthorn	2854	2850
**ν(C=O)**	Sea buckthorn, polysorbate	1744	1740
**νas(COO^-)**	Alginate	1607	1606
**δsc(–C–H) + νs(COO^-)**	Alginate	–	1469
**δ(–C–H) + νs(COO^-)**	Alginate	1457	1458
**νs(COO^-)**	Alginate	1415	1418
**δw(–C–H)**	Sea buckthorn, alginate	1377	1393
**δw(–C–H)**	Sea buckthorn, glycerol, polysorbate	1322	1330
**δϱ(–C–H)**	Alginate	–	1309
**ν(C–O)**	Alginate	–	1288
**ν(C–O)**	Alginate	–	1266
**ν(C–O)**	Alginate	–	1255
**ν(C–O) + δ(–C–H)**	Sea buckthorn	1240	1244
**ν(C–O) + δ(–C–H)**	Alginate	–	1236
**ν(C–O) + δ(–C–H)**	Alginate	–	1218

**ν(C–O) + δ(–C–H)**	Glycerol	1208	1196
**ν(C–O) + δ(–C–H)**	Alginate	–	1179
**ν(C–O–C)**	Alginate, glycerol, polysorbate	1100	1099
**ν(C–O)**	Alginate	–	1062
**ν(C–O)**	Alginate	–	1047
**ν(C–O–C)**	Sea buckthorn, alginate, glycerol	1032	–
**ν(C–O) + δ(C–OH)**	Glycerol	996	992
**ν(C–O)**	Alginate	–	944
**δ(O–H)**	Glycerol	924	–
**δ(–C–H)**	Glycerol	851	851
**δ(–C–H)**	Alginate, glycerol	819	819
**δ(–C–H)**	Alginate, sea buckthorn	718	720

Table S6. Kinetic modeling of polyphenol release from 3D-printed alginate emulgel patches.

**Kinetic model**	**Equation**	**Parameters**	**R^2**
Zero-order	$M_t = k_0 \cdot t + b$	k_0 = 1.376 %·h^{-1}; b = 25.74%	0.882
First-order	$\ln(100 - M_t) = \ln 100 - k \cdot t$	k = 0.0261 h^{-1}	0.843
Higuchi	$M_t = k_H \cdot \sqrt{t}$	k_H = 8.927 %·$h^{-0.5}$	0.860
**Korsmeyer–Peppas**	$M_t / M_\infty = k \cdot t^n$	**n = 0.221; k = 0.235 h^{-n}**	**0.866**
Weibull	$M_t / M_\infty = 1 - \exp[-(t/T_d)^b]$	b = 0.434; T_d = 36.55 h	0.835

Table S7. Structural recovery of the emulgel following high-shear treatment

Experimental stage	Time (sec.)	Viscosity (mPa·s) Mean ± SD (n = 3)	Structural recovery (%)
Initial structure (10 rpm)	0	11486.7 ± 11.5	100
High-shear treatment (100 rpm)	300	6751.7 ± 206.3	-
Recovery (10 rpm)	0	10003.3 ± 5.8	87.10
Recovery (10 rpm)	60	9988.3 ± 2.9	86.95
Recovery (10 rpm)	180	9975.0 ± 5.0	86.84
Recovery (10 rpm)	300	9796.7 ± 2.9	85.29
Recovery (10 rpm)	600	9785.0 ± 2.9	85.71
